# A methodology for exploring biomarker – phenotype associations: application to flow cytometry data and systemic sclerosis clinical manifestations

**DOI:** 10.1186/s12859-015-0722-x

**Published:** 2015-09-15

**Authors:** Hongtai Huang, Andrea Fava, Tara Guhr, Raffaello Cimbro, Antony Rosen, Francesco Boin, Hugh Ellis

**Affiliations:** 1 Department of Geography and Environmental Engineering, GWC Whiting School of Engineering, The Johns Hopkins University, Baltimore, MD USA; 2 Division of Rheumatology, Department of Medicine, Johns Hopkins School of Medicine, Baltimore, MD USA; 3 Present address: Division of Rheumatology, Department of Medicine, University of California, San Francisco, CA USA

**Keywords:** Scleroderma, Interstitial lung disease, Conditional random forests, Gene set enrichment analysis, Flow cytometry

## Abstract

**Background:**

This work seeks to develop a methodology for identifying reliable biomarkers of disease activity, progression and outcome through the identification of significant associations between high-throughput flow cytometry (FC) data and interstitial lung disease (ILD) - a systemic sclerosis (SSc, or scleroderma) clinical phenotype which is the leading cause of morbidity and mortality in SSc*.* A specific aim of the work involves developing a clinically useful screening tool that could yield accurate assessments of disease state such as the risk or presence of SSc-ILD, the activity of lung involvement and the likelihood to respond to therapeutic intervention. Ultimately this instrument could facilitate a refined stratification of SSc patients into clinically relevant subsets at the time of diagnosis and subsequently during the course of the disease and thus help in preventing bad outcomes from disease progression or unnecessary treatment side effects.

The methods utilized in the work involve: (1) clinical and peripheral blood flow cytometry data (**I**mmune **R**esponse **I**n **S**cleroderma, IRIS) from consented patients followed at the Johns Hopkins Scleroderma Center. (2) machine learning (Conditional Random Forests - CRF) coupled with Gene Set Enrichment Analysis (GSEA) to identify subsets of FC variables that are highly effective in classifying ILD patients; and (3) stochastic simulation to design, train and validate ILD risk screening tools.

**Results:**

Our hybrid analysis approach (CRF-GSEA) proved successful in predicting SSc patient ILD status with a high degree of success (>82 % correct classification in validation; 79 patients in the training data set, 40 patients in the validation data set).

**Conclusions:**

IRIS flow cytometry data provides useful information in assessing the ILD status of SSc patients. Our new approach combining Conditional Random Forests and Gene Set Enrichment Analysis was successful in identifying a subset of flow cytometry variables to create a screening tool that proved effective in correctly identifying ILD patients in the training and validation data sets. From a somewhat broader perspective, the identification of subsets of flow cytometry variables that exhibit *coordinated* movement (*i.e.*, multi-variable up or down regulation) may lead to insights into possible effector pathways and thereby improve the state of knowledge of systemic sclerosis pathogenesis.

**Electronic supplementary material:**

The online version of this article (doi:10.1186/s12859-015-0722-x) contains supplementary material, which is available to authorized users.

## Background

Much remains unknown regarding the etiology and pathogenesis of Systemic Sclerosis (SSc) and its treatment [1–6]. SSc pathogenetic processes involve development of fibrosis, vascular injury and autoimmune manifestations [6–9]. SSc patients are more susceptible than the general population to a variety of malignancies [10] and several possible clinical manifestations, notably interstitial lung disease (ILD) which is a major cause of death in SSc patients [11, 12]. In ILD, lung tissue becomes progressively hardened and replaced by scar tissue, resulting in loss of respiratory function. Lung transplantation is an option only in a minority of patients with severe ILD [13, 14]. As in the case of other autoimmune disorders, there are no curative therapies but only treatments aimed at halting progression towards end-stage disease [1]. Due to limited knowledge about the role of specific autoimmune effectors in the pathogenesis of SSc, conventional treatments such as immunosuppressant therapies are typically not targeted. They are also burdened by significant attendant morbidity and mortality [6, 15]. There are two main obstacles preventing a full understanding of SSc and the development of effective selective therapies. First, there is extreme heterogeneity in clinical manifestations among different SSc patients [12, 16]. The disease course is highly variable in terms of onset, timing, intensity of symptoms, patterns of organ involvement and response to therapy. The second major challenge derives from the occult nature of early immune effector pathways and the complex interaction of multiple humoral or cellular mediators, making the identification of the key drivers of clinical phenotypes difficult. Subsumed within this challenge is the difficulty in measuring and characterizing with precision the ongoing immune response [15, 17–23]. The current state of knowledge precludes any expectation of a near-term cure [4, 24, 25]. Clinical information is obtained from the Johns Hopkins Scleroderma Center, which actively follows more than 3000 patients. The biologic data is obtained via flow cytometry, a laser-based method of counting and characterizing selected cellular components in peripheral blood. This work describes the development of a hybrid generalizable method combining Conditional Random Forests and Gene Set Enrichment Analysis for identifying associations between flow cytometry data and interstitial lung disease. It is essentially a variable sub-setting method, but differs from existing approaches (*e.g.*, stepwise regression [26]) in its consideration of coordinated multi-variable up or down regulation. A clinically useful screening tool is developed that may aid in developing early assessments of the elevated risk of developing ILD, the activity of lung involvement and the likelihood to respond to therapeutic intervention.

## Methods

### Patients

The IRIS (Immune Response in Scleroderma Patients) cohort, from which the data set derived, was established to study longitudinally the ongoing immune response in relationship with the evolving clinical phenotype in a large number of well-characterized SSc patients. At each patient’s visit, multiparameter flow cytometry is performed to define the phenotype of circulating immune cells and is entered into a general database together with detailed clinical information. Patients evaluated at the Johns Hopkins Scleroderma Center were included in the IRIS cohort (after providing written informed consent) if they met the American College of Rheumatology preliminary criteria for the classification of SSc or had at least three of five features of CREST syndrome (Calcinosis, Raynaud’s phenomenon (RP), Esophageal dysmotility, Sclerodactyly, Telangiectasias) [16]. The Johns Hopkins Medicine Institutional Review Board approved the study.

### Clinical phenotyping

Demographic and clinical data including age, sex, ethnicity, smoking status, disease duration (from onset of Raynaud’s and from first non-Raynaud’s symptom), scleroderma subtype, specific organ involvement, use of immunosuppressive agents and autoantibody status (anti-topoisomerase-1, anti-centromere, anti-RNA polymerase III) were obtained at the time of each visit. Pulmonary involvement was determined based on abnormal pulmonary function tests (PFT), including measurements of forced vital capacity (FVC) and single-breath carbon monoxide diffusing capacity (DL_CO_), calculated according to the American Thoracic Society recommendations [27]. Spirometry values were referenced to those of the National Health and Nutrition Examination Survey [28]. Values for DL_CO_ were referenced to those reported in [28, 29]. Presence of ILD was defined as a FVC value less than 80 % of standardized predicted value [30, 31] – but we note that there exist other thresholds, most commonly, < 70 % [32, 33] – and confirmed in some cases by presence of fibrosis on high-resolution computerized tomography of the chest.

### Flow cytometry

Flow cytometry (FC) is a powerful tool used to analyze multiple characteristics of individual cells within heterogeneous populations [34–36]. Through more than seven decades of innovation [35, 37] flow cytometry has proven to be exceedingly useful in biological and medical studies, especially in the field of immunology [38–41]. Peripheral blood mononuclear cells (PBMC) were freshly isolated from whole blood through density-gradient centrifugation (Ficoll-Paque Plus, GE Healthcare). Cells were stained in staining buffer (PBS, 0.5 % BSA, 0.1 % sodium azide) at room temperature for 25 min. Activation, polarization, trafficking and naïve/memory state of CD4+ and CD8+ T cells were evaluated using four functional panels of markers. Within each functional panel, different T cell subsets are connected to each other through a hierarchical structure. Of the 158 SSc patients, 119 had a full complement of the data needed in these analyses.

Samples were analyzed with a standard manual gating strategy. Automated gating strategies for the analysis of complex multicolor flow cytometry data are of interest, however, there is not yet uniform agreement of what the best approach would be. In particular, the use of automated tools to analyze raw flow cytometry files is very difficult and prone to poor precision when acquisition of data is performed at different time points over a 5 year span, which is the case for our study. Moreover, reagents used are from different batches and the FACS machine undergoes different calibrations. Therefore, it is much better to conduct manual gating in order to accommodate for these variations. We favored a manual approach in this study in order to maximize precision. Details of the four panels and gating strategy are provided in Additional file 1: Section 1 (Flow Cytometry Details and Supplementary Results).

The FCS data, detailed description of the gating strategy and description of antibodies and fluorochromes can be found at https://flowrepository.org/id/FR-FCM-ZZLH. The IRIS data set and the R code developed in this work can be found in Additional file 2 (IRIS Data Set and R Code).

### Analytical tools – machine learning

Classification And Regression Trees (CART) is a modeling approach for classification (binary response) and regression (continuous response). CART is a non-parametric procedure (there is no reliance upon data distribution) comprised of a sequence of recursive tests, with the outcome of a current test determining the specifics of the next test and terminated by stopping criteria. The first test is to identify which FC variable is most important in accurately predicting ILD status and the value of that variable (from among all the values in the data set). There exist different metrics for importance depending upon whether CART is used for classification or regression [42]. An additional complication is how large to grow the tree (equivalently, how many splits to perform). A large tree may over-fit the data whereas a small tree may fail to capture important structure in the data. A balance between these two extremes is achieved through validation.

**Fig. 1:**
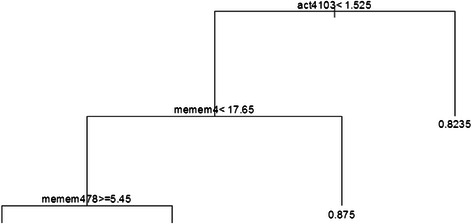
CART Result. CART output showing splitting variables and their respective values

Graphically, this gives rise to a tree-like structure shown in Fig. 1. For this example, FC expression act4103 at value 1.525 was identified as most important on the basis of greatest decrease in “node impurity” as represented by the GINI index [42]. SSc patients whose act4103 expression is less than 1.525 are split to the left branch, those with act4013 expression greater than or equal to 1.525 are directed to the right. The process is repeated (it is recursive) with the next most important variable identified as memem4 at value 17.65, and so on. In comparison with traditional regression, CART has advantageous attributes beyond being independent of data distribution: (1) CART is relatively insensitive to outliers in the input variables; (2) Stopping rules can be relaxed to over-fit the data - the training tree can then be pruned back to a level that maximizes validation performance; and, (3) CART can re-use variables in different parts of the tree and possibly uncover complex interdependencies between sets of variables.

The Random Forest (RF) modeling approach [26, 43] involves an ensemble of many regression or classification trees. Each tree in RF differs from CART in the following respects: (1) Random (bootstrap) sampling of the original data is used to create training subsets (as opposed to using the entire data set); and (2) The splitting variables at each node in a tree are randomly chosen from a subset of covariates as opposed to the pool of all covariates. The output from an RF model is the average of the performance from all of the regression trees generated.

The Conditional Random Forests (CRF) modeling approach is similar to RF in that it is also an ensemble of trees - but with the following modification - the variable selection process is separated from the splitting criteria and involves a hypothesis testing procedure. The null global hypothesis - all stimulus variables are independent of the response - is tested by examining the partial hypotheses that each stimulus variable is independent of the response. Only when the null global hypothesis is rejected does the variable selection process continue. This modification requires that each predictor variable selected as a splitting variable in each tree to be strongly associated with the response variable through hypotheses testing under an unbiased conditional inference framework [44]. This process exploits the discriminatory power of predictor variables when numerous covariates are highly correlated [45, 46]. Strobl et al. [47] showed that variable importance measures (VIM) based on this conditional permutation scheme above better match the coefficients associated with greatest predictor discriminatory power and that VIM stability was improved over that of the unconditional importance approach. We always performed multiple CRF runs (typically 20 - each initiated by a randomly generated seed) to enable computation of a mean variable importance list VIL (CRF results are stochastic – the VIL from one run to another can change).

Support Vector Machines (SVM) [48] is a non-probabilistic binary linear classifier which takes predictor data as input; the output is a prediction function. In this application, flow cytometry data are the inputs with ILD status (0 or 1) as the prediction output. FC data are represented as points in space, with prediction arranged (“mapped”) into categories (0 = non-ILD; 1 = ILD) separated by as large a distance as possible. Extensions to nonlinear partitioning are accomplished by expanding the predictor variable space through so-called kernel functions [49, 50].

We did not pursue methods that involved composite variables (*e.g.*, weighted sums of flow cytometry expressions as would be the case in Principal Component Analysis [51]) because of the difficulties presented when attempting to hypothesize biological interpretations of composite predictors. The performance measures used to evaluate the methods were mean absolute and mean squared predictions errors and Receiver Operating Characteristics (ROC) curves [52–55].

### Analytical tools - gene set enrichment analyses

**Fig. 2:**
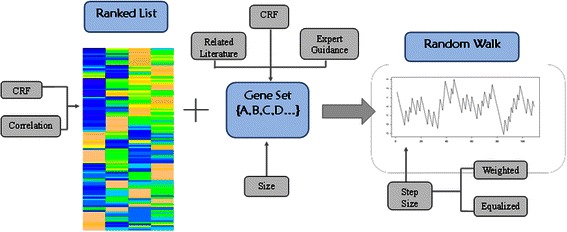
GSEA Schematic. Hybrid Gene Set Enrichment Analysis modeling approach. Conditional Random Forests is first run to generate a Variable Importance List. That list, along with a ranked list of FC variables (ranked by correlation with the phenotype) constitute the input to GSEA. FC sets are created top-down from the Variables Importance List

Gene Set Enrichment Analysis [56, 57] was motivated by gene expression studies which showed that analyses involving the study of only one gene at a time were of limited usefulness. Approaches examining sets (or cassettes) of genes showed greater ability to identify meaningful associations between gene expression and disease state. Gene sets became “FC sets” in this work, with ILD being the phenotype. We form FC sets using the variable importance list (VIL) obtained through CRF. The VIL contains FC variables ordered by marginal greatest decrease in prediction accuracy [47] – our FC sets are formed from the top down (*i.e.*, FC set size *G* contains the top *G* variables in the VIL). Having identified an FC set, a random walk is then performed. This process, shown in Fig. 2, involves first estimating correlation coefficients between response ILD and all (112) flow cytometry variables, then ranking them from largest positive to largest negative. The FC set is then moved down the correlation-ranked list from top to bottom, one variable per step, and recording a running sum for each step. If a variable in the correlation ranked list is encountered that is in the FC set, the following quantity is added to the running sum:

$$ \sqrt{\frac{N-G}{G}} $$

otherwise, subtract from the running sum

$$ \sqrt{\frac{G}{N-G}} $$

where, G is size of the FC set and N is the total number of FC variables (112). Enrichment Score supremum retains its original GSEA meaning – evidence of strong coordinated movement (up or down regulation) of expressions (Subramanian et al. [57]). The ‘best’ FC set size is associated with the largest ES.

Significance testing of ES values is accomplished empirically through permuting phenotype [57]. For a given FC set size (with enrichment score ES*) and correlation-ranked FC variable list, GSEA is performed 10,000 times – each time with the phenotype permuted – and the resulting ES supremum recorded. The p-value is the number of ES scores larger than ES*, divided by 10,000. We experimented with different numbers of permutations (up to 1,000,000) and settled on 10,000 as a reasonable balance of significance level and computational effort.

Note that our approach involves *two* ranked lists. First is the CRF variable importance list (VIL) used to form FC sets. Alternatives to CRF_VIL for creating FC sets are discussed in Results - *Alternative Rules for Creating FC Sets*. Second is the ranked correlation list in GSEA (signed Pearson product moment or Spearman correlation with phenotype). An alternative to signed correlation is an absolute value correlation list, whereupon only up-regulation in the random walk is possible.

### Analytical tools - stochastic simulation

CRF is a poor out-of-sample (*i.e.* out-of-bag, OOB) predictor in this application (described later in Results) which necessitated an alternative method for prediction. This gave rise to the development of screening tools via stochastic simulation. The screening tools are very simple in construction. First, a subset of FC variables is chosen to be included in the screening tool. This process can be very computationally challenging in that we potentially have *N* FC variables (*N* = 112) with which to construct screening tools, but no a priori knowledge of how many variables and which variables should be included in the screening tool. A conservative but very computationally expensive approach would involve full combinatorial expansion, that is, we would evaluate screening tools comprised of $$ \left(\begin{array}{c}\hfill N\hfill \\ {}\hfill 1\hfill \end{array}\right),\left(\begin{array}{c}\hfill N\hfill \\ {}\hfill 2\hfill \end{array}\right),\dots \kern0.5em ,\kern0.5em \left(\begin{array}{c}\hfill N\hfill \\ {}\hfill N\hfill \end{array}\right) $$ FC variables*.* As explained below in GSEA results, the computational challenge is, however, considerably lessened (the best screening tools were found with N =27 and three to six FC variables chosen for all screening tools). Nonetheless there now remains a critical randomization step. A patient is declared ILD if *any* of their FC expressions is above a positive or below a negative standardized threshold. Standardized threshold deviates are computed using the FC expression ranges obtained from the IRIS data. In every screening tool realization (involving *k* FC variables) 2 *k* thresholds (one lower, one upper threshold per variable) are randomly generated using a uniform generator [58] which is a reasonable way to explore a large unknown parameter space.

We experimented with different metrics to assess screening tool performance: (1) the ratio of number of predicted ILD patients to the sum of correctly predicted ILD and incorrectly predicted no-ILD patients; (2) the ratio of the number of predicted ILD patients to the true number of ILD patients (*i.e.*, the True Positive Rate); (3) The product of (1) and (2) (which penalizes screening tools with good ILD prediction but poor no-ILD prediction); and (4) One minus the fraction of total misclassified patients (the Overall Misclassification Rate, OMR) that equally weights both forms of misclassification. We decided on a two-level metric (OMR with TPR used break ties if necessary) because we had discovered that in some situations best screening tools were not unique. Using the guidelines provided in [59] we randomly divided our IRIS data (119 patients) into two groups: a training data set (79 patients) and a validation data set (40 patients).

## Results

### Data mining

Five classification methods were tested using training data (Classification and Regression Trees - CART), Random Forests (RF), Conditional Random Forests (CRF), Support Vector Machines (SVM) and a mean-only model) using 112 FC expressions as predictor variables and ILD as response. The mean-only model simply uses the mean value of the response in the training data as the prediction. Table 1 shows the mean absolute and mean squared errors of the five approaches.

**Table 1:** Mean absolute and mean squared errors

	MAE	MSE
CART	0.471	0.356
RF	0.482	0.246
CRF	0.492	0.248
SVM	0.502	0.253
mean	0.502	0.254

**Fig. 3:**
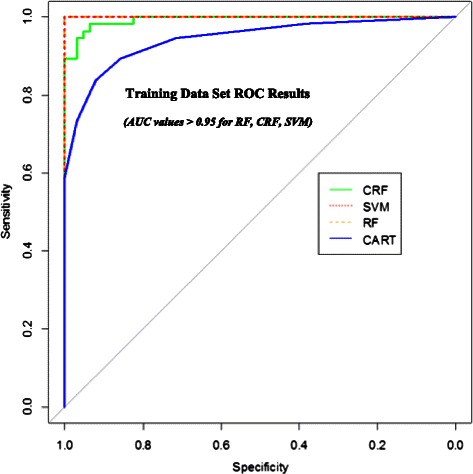
ROC results for CRF, RF, SVM and CART. Receiver operating Characteristic Curve results for Conditional Random Forests, Random Forests, Support Vector Machines and Classification and Regression Trees

RF and CRF perform best with the training data (by a small amount) but their differences in MAE and MSE are not statistically significant (p-value = 0.0996 for MAE and p-value = 0.785 for MSE). The mean-only result confirmed our understanding that this statistical estimation problem is very flat (*i.e.*, no single FC variable or small subset of variables is highly associated with ILD status). From their Receiver Operating Characteristic (ROC) curves, performance (Fig. 3) RF, SVM and CRF emerged as the most effective classifiers. All consistently yielded AUC (Area Under Curve) values of greater than 0.95 for the training set data.

Conditional Random Forest (CRF) was eventually chosen over RF and SVM for several reasons. First, and most importantly, the permutation computing scheme for variable importance measures (VIMs) in CRF theoretically provides a potentially better method for identifying truly relevant predictors [47, 60]. Specifically, it enforces the requirement that each predictor variable that is selected as a split variable in each tree must be strongly associated with response variables (through hypotheses testing under an unbiased conditional inference framework). This is a robust way of enhancing the discriminant power of a predictor variable. In contrast, the VIMs of RF can be unstable and suffer from ‘correlation bias’ due to effects related to predictor correlation, including: (1) VIMs are not necessarily aligned with the discriminant power of the stimulus variable; (2) the size of the group of correlated variables can have a pronounced effect [45, 46]; and, (3) VIMs did not directly reflect the coefficients in the generating model [61]. Using this conditional importance measure, Strobl et al. [47] showed that VIMs based on the conditional permutation scheme better reflect the pattern of the coefficients associated with predictor discriminant power and that the variability was lower than that of the unconditional importance within each level of mtry (where mtry is the parameter in R that sets the number of variables to include in any random tree). Second, the contingency tables of the fitted CRF, RF and SVM models suggested that RF and SVM might be over-fitting. CRF misclassified 5 patients out of 79 whereas RF and SVM had 100 % predictive accuracy. Third, the Enrichment Scores (ES) based on the variable importance list drawn from CRF were always larger than those of RF and SVM regardless of configuration settings, including the number of trees and the number of covariates randomly selected to split the node in each tree of the RF model [60]. The results are shown in Table 2. In application, the anticipated benefits of conditional variable selection in CRF (through specifying conditional = TRUE in the R package “party” [60]) did not materialize. Extensive testing with and without conditioning produced virtually identical variable importance lists. We eventually chose not to invoke conditioning because it is computationally very expensive.

**Table 2:** RF and CRF parameters and best enrichment scores

mtry	ntree	RF	CRF
5	1000	21.77	23.43
11	1000	20.76	22.34

### Gene set enrichment analysis

**Fig. 4:**
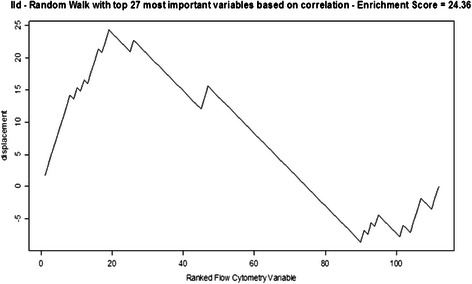
Example random walk. Gene Set Enrichment Analysis random walk for a flow cytometry set comprised of 27 variables

**Fig. 5:**
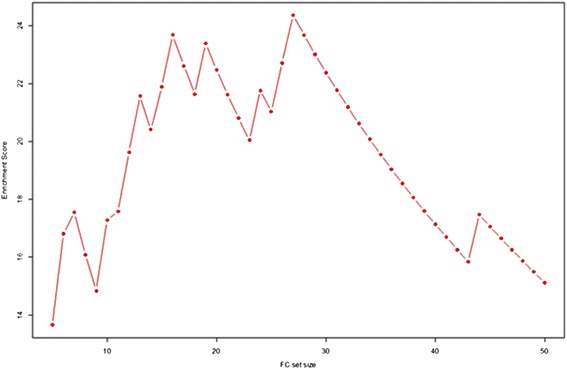
Plot of enrichment score versus FC set size. Gene Set Enrichment Analysis enrichment score plotted as a function of FC set size (obtained top-down from the CRF Variable Importance List)

**Fig. 6:**
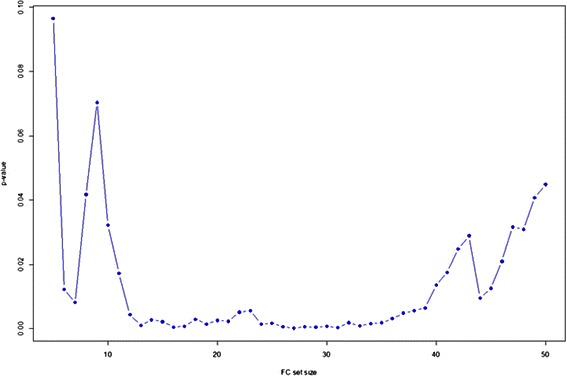
Statistical significance levels of the ES values shown in Fig. 8. Statistical significance levels for the enrichment scores shown in Fig. 8

FC set sizes (*G*) from 3 to 50 were tested (for a set size of *G* and 112 FC variables in total, the random walk increases by $\sqrt{\frac{112-G}{G}}$ if a variable in the correlation ranked list is in the FC set and decreases by $\sqrt{\frac{G}{112-G}}$ otherwise). The results are shown in Fig. 4. This is representative random walk behavior for those FC sets that subsequently performed well in screening tool training and validation, exhibiting strong up-regulation producing the ES, then extended decline in displacement and finally a moderate increase in displacement for FC variables negatively correlated with ILD. The maximum displacements from zero (the enrichment scores, ES) for all random walks for all *G* are plotted in Fig. 5. FC set size (27) had the highest Enrichment Score (suprema of all the random walks). Figure 6 shows the statistical significance levels for each of the ES values. For the FC sets of interest (*i.e.*, those with the largest ES values) p-values are typically less than 0.0001. Table 3 shows the variables included in FC27, their mean VI values (*i.e.*, act4103 is responsible for the greatest decrease in classification error; pol8cc5 follows, and so on) and their correlations with the phenotype. Note that the VIL contains FC variables at the top of the list that are both positively and negatively correlated with phenotype.

**Table 3:** Best FC set variables and definitions

FC Variable	Mean VI	Correlation
act4103	2.28E-03	0.233977426
pol8ccr5	2.24E-03	−0.286711053
act425tot	1.17E-03	0.220684788
act425lo	1.17E-03	0.22023626
pol8th17	1.00E-03	−0.157540769
mememra4	9.28E-04	0.248352001
act8103	8.45E-04	0.163452972
pol8ccr5cxcr3neg	7.95E-04	−0.226940337
pol8ccr4	4.24E-04	−0.157290699
act425103	4.00E-04	0.201942746
pol8th1th2ratio	3.77E-04	0.126269489
act810371	3.09E-04	0.21094877
memem4	3.06E-04	0.141766889
pol8x3r4ratio	2.41E-04	0.041171241
memem478	2.36E-04	0.122724989
mememra4k	1.73E-04	0.155600977
pol8th2	1.69E-04	−0.106939105
memem878	1.60E-04	−0.123626487
mememra478	1.19E-04	0.206671725
memem48	9.04E-05	0.043826522
act4dr	7.40E-05	−0.139383714
memem8	3.43E-05	−0.100496411
memcm478	−4.50E-06	0.094476259
memcm4k	−2.96E-05	0.169922925
act8dr	−4.01E-05	−0.179623785
traff4ccr3	−4.56E-05	0.138343962
pol4ccr6	−5.23E-05	0.181748831

#### Alternative rules for creating FC sets

**Fig. 7:**
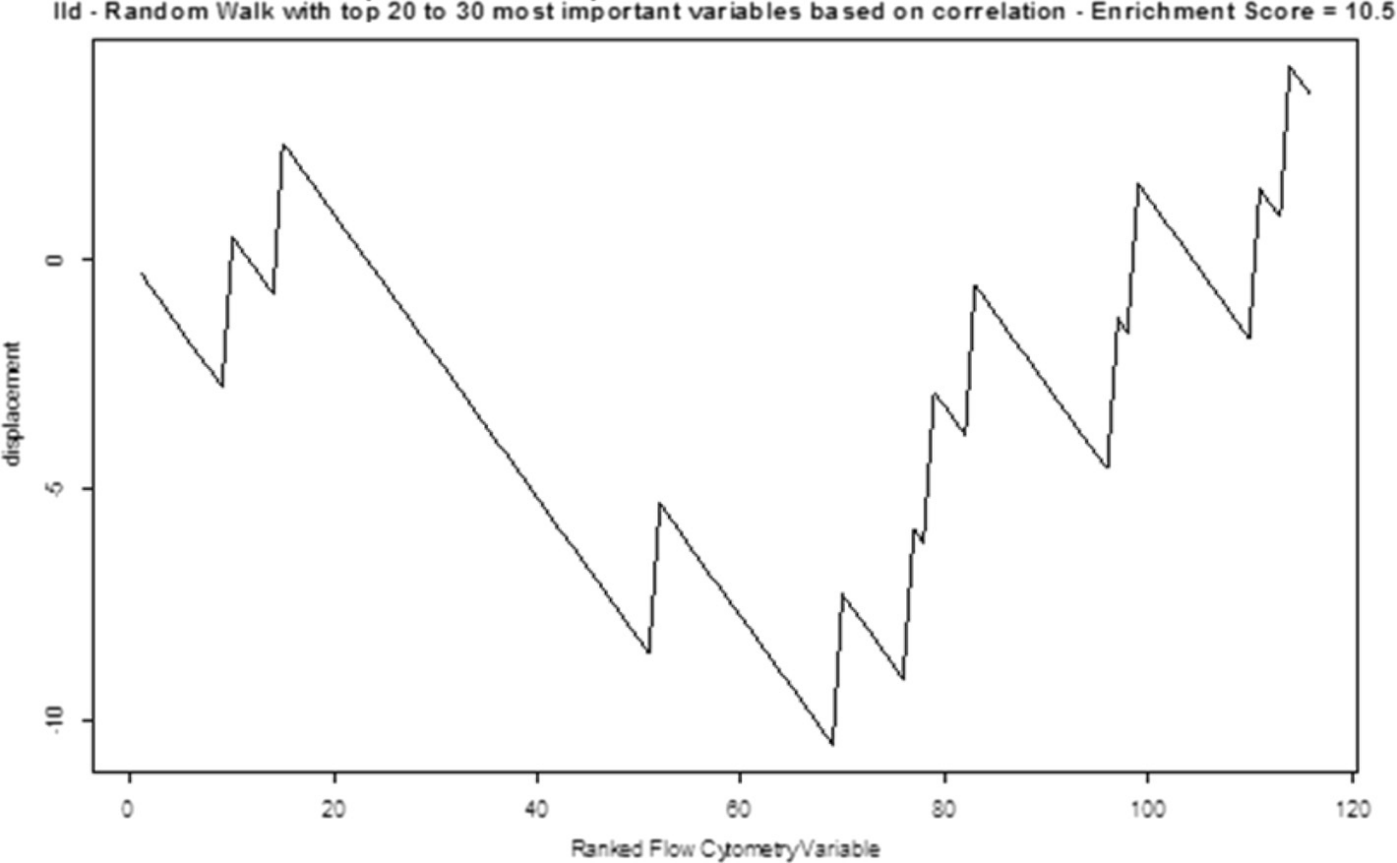
Random walk for the FC set comprised of the 20th to 30th highest ranked variables. Random walk for the FC set comprised of the 20th to 30th highest ranked variables in the Conditional Random Forest variable importance list

**Fig. 8:**
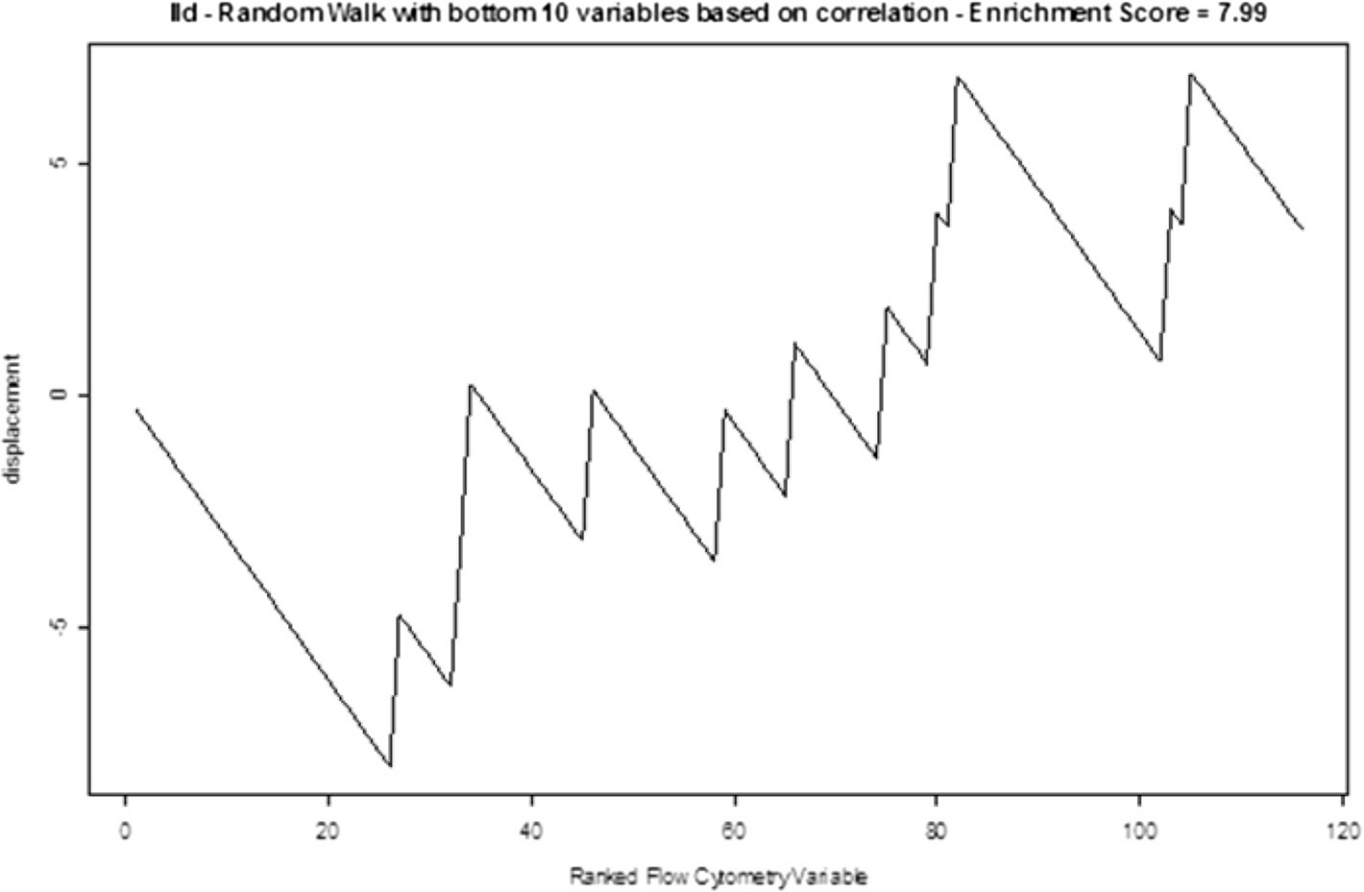
Random walk for the FC set comprised of the bottom ten ranked variables. Random walk for the FC set comprised of the bottom 10 ranked variables in the Conditional Random Forest variable importance list

**Fig. 9:**
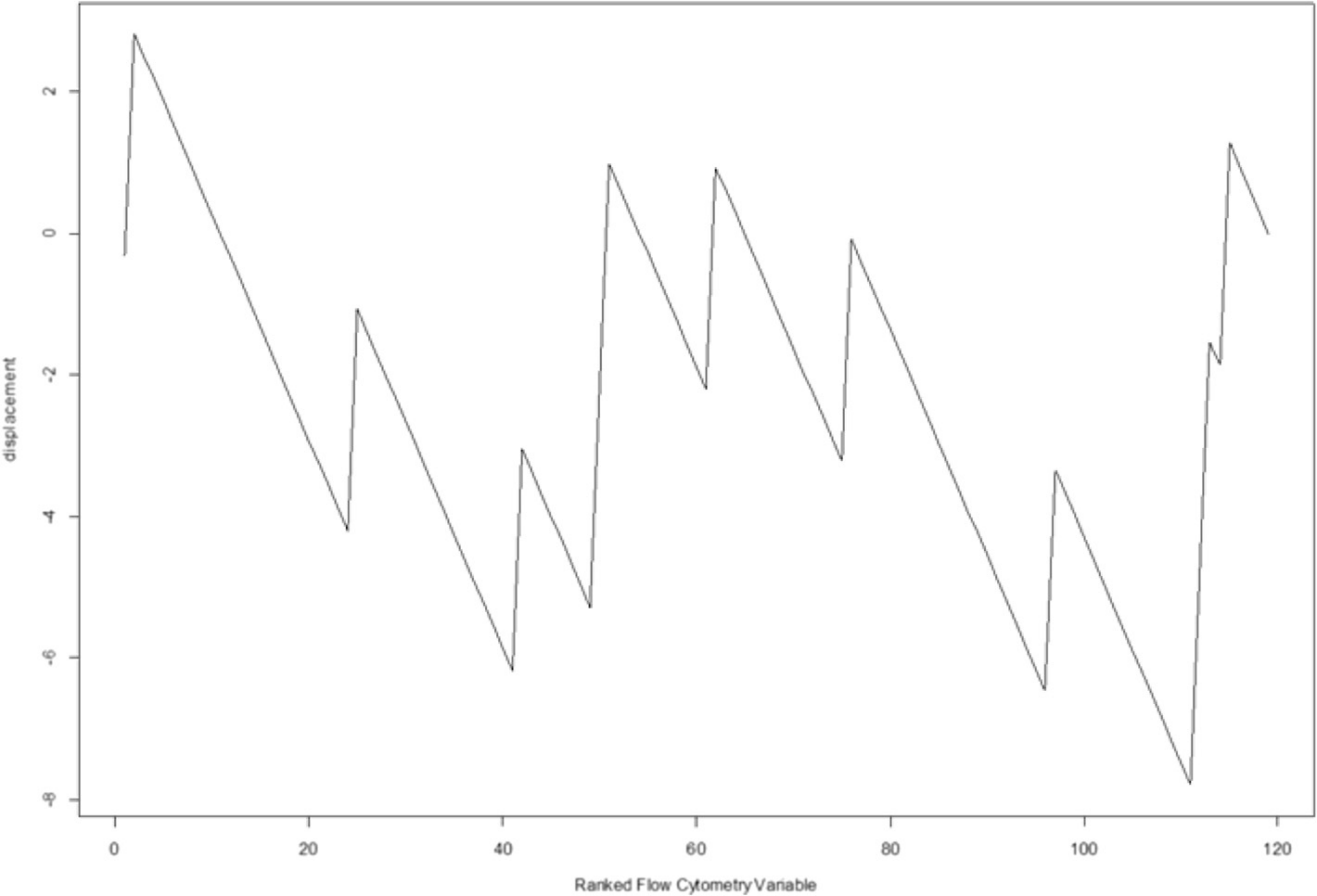
Random walk for the CD4 FC set. Random walk for the CD4 FC set, comprised of possibly important markers

**Fig. 10:**
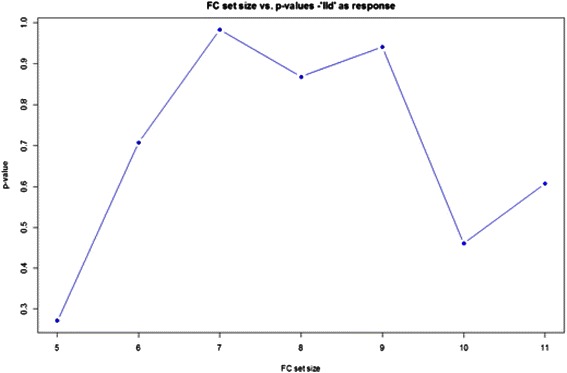
Statistical significance of the ES vales in Fig. 13. Statistical significance of the enrichment scores for the CD4 GSEA results

We further experimented with GSEA by modifying the rule originally created for constructing FC sets. Instead of working from the top down in the VIL, we formed FC sets from variables with lower-ranking variable importance values. Figure 7 shows the random walk that resulted from an FC set comprised of the 20th to 30th ranked variables in the VIL. Figure 8 shows the random walk using an FC set comprised of the bottom ten ranked variables of the VIL. Low enrichment scores and very irregular walks were the result. Another modification abandoned the CRF-created VIL altogether for creating FC sets and instead used the sets in Table 4 comprised of possibly important markers, drawn from prior experience (in essentially a hypothesis generation process). The random walk for the CD4 FC set is shown in Fig. 9. It has very irregular structure and a relatively low enrichment score. Figure 10 shows the significance levels for FC set sizes 5 to 11. No FC sets had statistically significant enrichment scores (p > 0.25). CD8 results were comparable.

**Table 4:** Alternative FC sets

CD4	CD8
pol4th1	act8103
pol4th2	pol8cxcr3
pol4th17	pol8ccr4
pol4th1th17	pol8ccr6
act425lo	act8dr
act425hi	traff8cxcr6
act4dr	traff8ccr10
traff4ccr10	memnaive8
traff4cxcr6	mem8cmefratio
memnaive4	mem8ememraratio
mem4cmefratio	memcd8k

### Randomized screening tool design and testing for ILD vs. no-ILD classification

**Fig. 11:**
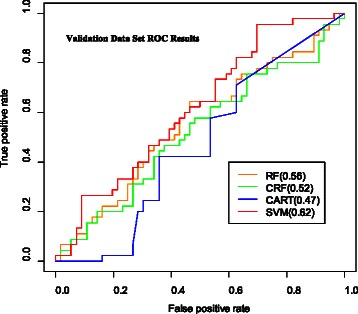
CRF, RF, CART, SVM Predictive Performance – ROC. CRF and the other machine learning methods have poor predictive performance

In contrast to good training performance, CRF is a poor out-of-sample (*i.e.* out-of-bag, OOB) predictor in this application as shown in Fig. 11 (the predictive performance of CRF is no better than predicting ILD status by flipping a coin) – which is why we created screening tools in a different manner – via stochastic simulation. Our design guidelines were simplicity and parsimony. Randomly generated bounds for each FC variable were derived from analyses using FC variable ranges obtained from the training set data. We then addressed the issue of which subsets of variables (from the set of 27 ‘best’ variables identified through GSEA) should be used to construct screening tools. Experiments were conducted to see whether full combinatorial expansion was necessary, that is, did we need to evaluate tools with $$ \left(\begin{array}{c}\hfill 27\hfill \\ {}\hfill 1\hfill \end{array}\right) $$ through $$ \left(\begin{array}{c}\hfill 27\hfill \\ {}\hfill 27\hfill \end{array}\right) $$ components (134,217,727 possible combinations of variables; recall also that for each combination, many random threshold realizations are generated). Extensive testing using full combinatorial expansion (which consumed about 200,000 service units of compute time on a very powerful parallel computer – Kraken [62]; a typical run involved simultaneous execution of several thousand R instances) showed that the best performing screening tools (those with the lowest OMR) were consistently comprised of at least three and no more than six FC variables, therefore all subsequent analyses involved screening tools comprised of $$ \left(\begin{array}{c}\hfill 27\hfill \\ {}\hfill 3\hfill \end{array}\right) $$ through $$ \left(\begin{array}{c}\hfill 27\hfill \\ {}\hfill 6\hfill \end{array}\right) $$ (*i.e.*, 2,925 + 17,550 + 80,730 + 296,010 = 397,215) combinations of FC variables. The best performing tool at this stage of the analyses had an overall training misclassification rate of 18.98 % (15 patients misclassified out of 79; 11 ILD, 4 no-ILD).

**Fig. 12:**
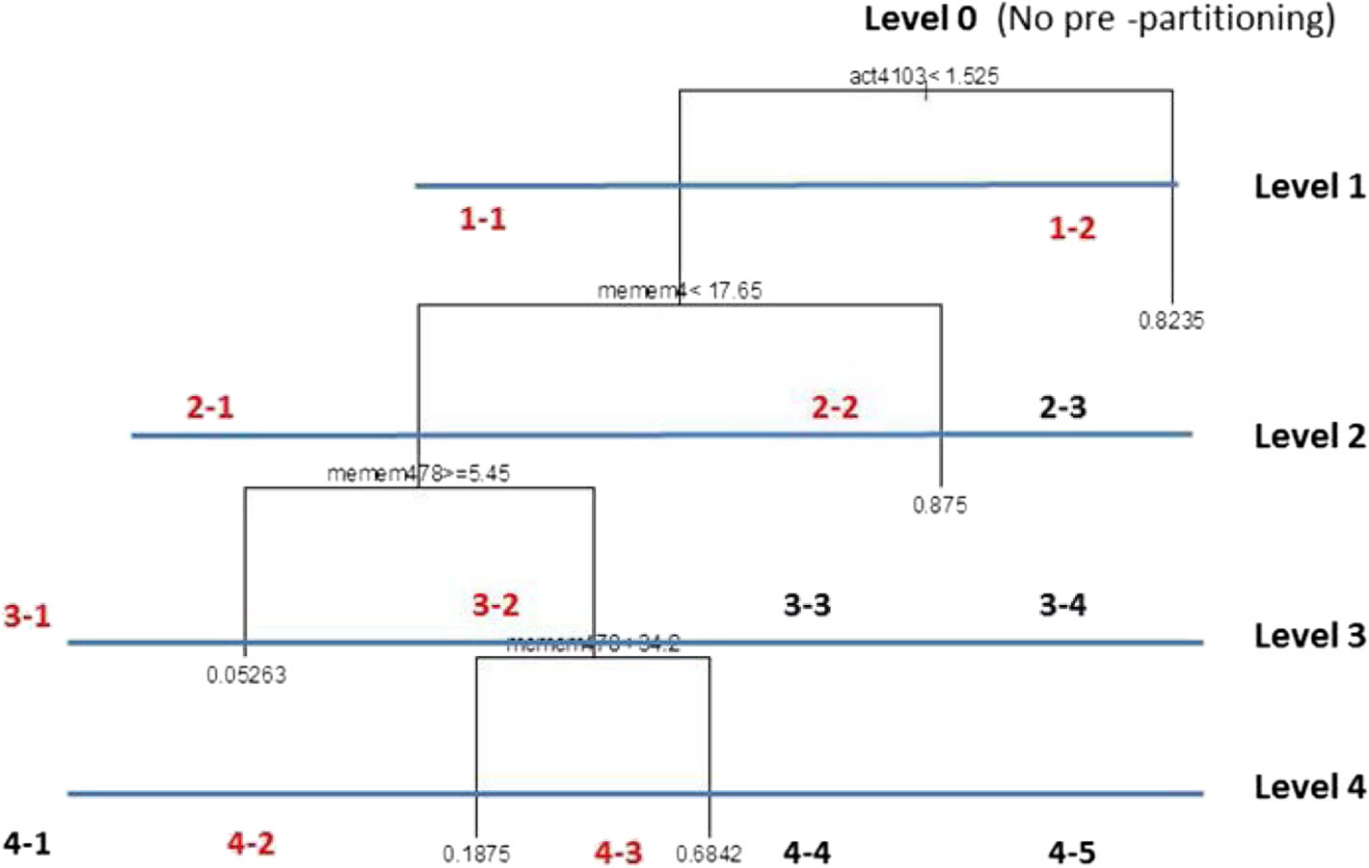
CART Pre-partitioning result. Classification and Regression Trees pre-partitioning result. Patients are divided into groups, with screening tools identified for each group (as opposed to finding best screening tools for the entire set of training patients)

A refinement to screening tool design involved grouping (*i.e.*, pre-partitioning) the 79 training patients into sub-groups using CART and generating screening tools separately for each sub-group. CART automatically selects FC (“splitting”) variables and associated numerical values of those variables that most successfully group patients by ILD status. Figure 12 shows the CART pre-partitioning results. We note that this adds considerably to the computing burden – best screening tools now must be identified for multiple groups within levels (with the same splitting criteria subsequently used to pre-partition patients into groups for validation). Randomized screening tool design was then performed for each subgroup (there are 14 shown in total in Fig. 12, but six are child nodes whose parents have OMR = 0; these six node ID’s are shown in black). This method substantially improved screening tool performance. The pre-partitioned OMR results using training data are shown in Table 5 (MC = Misclassified).

**Table 5:** Pre-partitioned OMR results

CART Node	# ILD	# NoILD	# MC	# ILD MC	# NoILD MC	OMR
Level 0	38	41	15	11	4	0.1878
1-1	24	38	11	9	2	0.1774
1-2	14	3	0	0	0	0.0
Level 1	38	41	11	9	2	0.1392
2-1	17	37	9	8	1	0.1667
2-2	7	1	0	0	0	0.0
Level 2	38	41	9	8	1	0.1139
3-1	1	18	1	0	1	0.0526
3-2	16	19	5	2	3	0.1429
Level 3	38	41	6	2	4	0.0759
4-2	3	13	1	0	1	0.0625
4-3	13	6	0	0	0	0.0
Level 4	38	41	1	1	1	0.0127
Parent Node > Child Node; 1–2 > 2–3 > 3–4 > 4–5; 2–2 > 3–3 > 4–4; 3–1 > 4-1						

Excepting the fourth level (where only one patient of 79 was misclassified) there was considerably more misclassification of ILD than no-ILD patients. The result that only one patient was misclassified at the fourth level of pre-partitioning is suggestive (but not proof) of over fitting, which is addressed below in validation. Additional training filter details are provided in Additional file 1: Sections 2 and 3.

### Validation

**Fig. 13:**
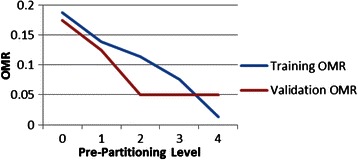
Screening tool performance: Training versus validation error Screening tool training and validation performance showing that over fitting in training was occurring. The best training screening tools are not the best performing validation screening tools

The best training screening tools were then validated using FC data from the 40 patients whose data was not used in training. Without pre-partitioning (Level 0 in Fig. 13) the overall correct ILD classification rate was 82.5 % (seven patients misclassified out of 40; 3 ILD, 4 no-ILD). Pre-partitioning the validation patients (using the CART-derived variables and splitting levels developed for the training data) increases correct validation classification to 95 % after two levels of pruning (two patients misclassified out of 40). This indicates that over fitting was occurring in training for the deepest pre-partitioning level (the training and validation curves indeed cross) as Fig. 13 attests. Notable also is the similar OMR performance between training and validation for no pre-partitioning (Level 0). As a corollary, the existence of training over-fitting is also indicated by the result that the best-performing training screening tools are not the best-performing validation screening tools. Additional validation filter details are provided in Additional file 1: Sections 2 and 3.

## Discussion

### Hybrid approach

Several different data mining techniques proved effective in classifying the ILD status of SSc patients in the training data set, given their FC data as predictors. CRF was eventually chosen based mostly on its capability to deal with the effects of correlated predictor variables. The ES values associated with CRF-VIL FC sets (formed from the group of variables at the top CRF variable importance list) were consistently statistically significant based on the GSEA permutation test. There exists a unique FC set size associated with an Enrichment Score supremum. Larger FC set sizes did not necessarily lead to greater enrichment scores. These methods, however, did not perform well in prediction (*i.e.*, validation) which necessitated the development of an alternative method for screening tool design.

### Other machine learning methods

There exist other machine learning approaches that have not yet been evaluated. For example, Bayesian confidence propagation neural network (BCPNN) was shown to be effective in classification in some medical science applications [63, 64]. Also, the selection of phenotype can significantly affect model performance. This was revealed in poor classification and GSEA performance when using pah_45 as phenotype.

### VIL robustness

In our ‘top-down’ analyses (forming FC sets beginning at the top of the ranked correlation list in GSEA) all random walks were up-regulated with relatively strong enrichment scores. To test the robustness of this result, we instead constructed FC sets comprised of variables with lower ranked variable importance values, and in one case, formed FC sets beginning at the bottom of the ranked list. These tests typically yielded irregular down-regulated random walks with much smaller enrichment scores. There is strong evidence therefore that the CRF-derived variable importance list, and forming FC sets top-down from this list, plays an important role in good GSEA performance. We note as well the possibility to create variable importance lists (or their equivalent) with methods other than CRF.

### The GSEA ranked list

Thus far, the ranked lists of the GSEA test were based on sorted, signed correlation coefficients. This is the original and most common approach in GSEA, but others exist, for example, the absolute value of correlation coefficients could serve as the ranked list. It follows in that case that all random walks would be up-regulated.

#### GSEA – FC set determination

In the original genomics applications, gene sets for GSEA were typically identified through some form of hypothesis generation procedure – typically based on biological insight (our rough equivalent would be insight into possible SSc effector pathways). A reasonable alternative here is to define FC sets based on FC variables considered to be potentially important biomarkers. Two such FC sets (CD4-based and CD8-based) were created and tested through GSEA. Performance was poor when compared with the hybrid CRF-GSEA approach, but this exercise was very limited in scope.

### GSEA – permutation test

Gene Set Analysis (GSA) in general can be divided into two major types based on the permutation schemes used in their statistical tests: class label randomization and gene randomization [65]. The GSEA algorithm in this research, SAFE and SAM- CS [66, 67] belong to the first category, while PAGE, T-profiler and Random Set [68–70] are classified as gene-randomization. An important factor in assessing the suitability of an approach is sample size (our data set contains 119 patients). Our results were robust with respect to the number of permutations used to estimate p-values. The alternative approach (gene-randomization) is to permute FC variables, but this will lead to nonconservative significance levels, *i.e.* smaller p-values resulting in more false positives, because this approach does not account for stimulus (predictor) variable correlation [57].

### Randomized screening tool design

Randomized screening tool design is a novel and, we believe, generalizable mathematical tool used for phenotype classification. Its non-parametric nature allows application in many other settings. This approach allows for predictor variables and responses to be continuous, binary or categorical. We also note that the need for creating the randomized screening tool arose because CRF performed poorly in prediction. If another machine learning (or other) method performed well in prediction, then the randomized screening tool would be unnecessary.

While training screening tools performed very well when classifying the entire data set (OMR = 0.1898), the added step of pre-partitioning patients using CART (and finding best training screening tools specific to each CART group) significantly improved screening tool performance. The splitting criterion used in CART appears to be a good starting point for identifying subpopulations of SSc patients. Our training screening tool experiments consistently resulted in best screening tools having three to six components, with five most often.

### Validation

Validation was successful with a correct classification rate of 82.5 % for the entire validation Data set (40 patients), increasing to 95 % with CART pre-partitioning. There exists a reasonable balance between training and validation error. We would expect that as more data becomes available, screening tool performance will continue to improve. This suggests another potentially important role for our approach in better understanding the progression of disease. We posit the scenario in which FC profile characteristics may change with disease progression and that these changes could be captured – reflected in changes in screening tool design and performance. Selected FC variables and their expressions could be used as a basis for partitioning patients into disease progression states, with corresponding state-specific screening tool designs.

### Biological interpretation

In FC27, the two FC variables (pol8ccr5cxcr3neg, pol8ccr5cxcr3) identify Th1 polarized CD8 T cells. The first (lacking CXCR3) is “protective”; the second (CXCR3) is a “risk factor” for ILD. CXCR3 is a chemokine receptor which has been shown to direct inflammatory cells inside target tissue and drives acute inflammation (synovial tissue in rheumatoid arthritis, liver in autoimmune hepatitis, etc.). The variables memem8, mememra87, memcm878 and memcm4 belong to the T cell memory subset. It appears that ILD status is associated with a shift of the CD8 T cells towards the activated effector memory/terminally differentiated state. This is in keeping with the pro-inflammatory polarized status observed.

## Conclusions

IRIS flow cytometry data provides useful information in assessing the ILD status of SSc patients.

Our hybrid analysis approach is: (1) Conditional Forest classification to produce a variable importance list; (2) Gene Set Enrichment Analysis to identify ‘best’ sets of flow cytometry variables; (3) random screening tool generation and training to identify best screening tools on the basis of their overall misclassification rate performance; and (4) validation. With the IRIS data set, it proved successful in predicting SSc patient ILD status with a high degree of success. The identification of subsets of flow cytometry variables through our methodology may also lead to insights into possible effector pathways and thereby improve the state of knowledge of systemic sclerosis pathogenesis. HRCT confirmation of patient ILD status is an important next step in developing additional confidence with our approach (and the appropriateness of the commonly used 80 % FVCstpp threshold for presumptive ILD determination). The approach described in this report perhaps could be used as a new screening tool to identify relevant variables that account for phenotypes in unrelated diseases.

## Additional files

Additional file 1:
**Flow Cytometry Details and Supplementary Results.** (DOCX 1.93 MB)
Additional file 2:
**IRIS Data Set and R code.** (ZIP 291 KB)

## Abbreviations

BCPNN: Bayesian Confidence Propagation Neural Network
CART: Classification and regression trees
CD4: Helper T-cell
CD8: Cytotoxic T-cell
CRF: Conditional random forests
CT: Computed tomography
CXCR3: CXC chemokine receptor 3
ES: Enrichment score
FC: Flow cytometry
FVCstpp: Uniformly standardized percent predicted Forced Vital Capacity
GLM: Generalized linear model
GSEA: Gene set enrichment analysis
HRCT: High resolution computed tomography
ILD: Interstitial lung disease
IRIS: Immune response in scleroderma
MAE: Mean absolute error
MSE: Mean squared error
OMR: Overall misclassification rate
OOB: Out-of-bag
PAGE: Parametric analysis of gene set enrichment
PCA: Principal component analysis
RF: Random forests
ROC: Receiver operating characteristics
SAFE: Significance analysis of function and expression
SAM-GS: Significance Analysis of Microarray to Gene-Set analyses (SAM-GS)
SSc: Scleroderma
SVM: Support vector machines
Th1: Cytokine signature; one of two distinct subclasses of immune response
TPR: True positive rate
VIL: Variable importance list
VIM: Variable importance measure

## References

[1] Winstone TA, Assayag D, Wilcox PG, Dunne JV, Hague CJ, Leipsic J, Collard HR, CJ. Ryerson: Predictors of mortality and progression in scleroderma-associated interstitial lung disease: A systematic review. Chest, 2014 10.1378/chest.13-2626 24576924

[2] Varga J. http://www.uptodate.com/contents/prognosis-and-treatment-of-interstitial-lung-disease-in-systemic-sclerosis-scleroderma#H1. 2014.

[3] Wahren-Herlenius M, Dorner T (2013). Immunopathogenic mechanisms of systemic autoimmune disease. Lancet.

[4] Roth MD, Tseng CH, Clements PJ, Furst DE, Tashkin DP, Goldin JG, Khanna D, Kleerup EC, Li N, Elashoff D, Elashoff RM, G. Scleroderma Lung Study Research (2011). Predicting treatment outcomes and responder subsets in scleroderma-related interstitial lung disease. Arthritis Rheum.

[5] Strange C, Seibold JR (2008). Scleroderma lung disease: If you don’t know where you are going, any road will take you there. Am J Respir Crit Care Med.

[6] Boin F, Rosen A (2007). Autoimmunity in systemic sclerosis: current concepts. Curr Rheumatol Rep.

[7] Rosen A, Casciola-Rosen L (2009). Autoantigens in systemic autoimmunity: critical partner in pathogenesis. J Intern Med.

[8] Gabrielli A, Avvedimento EV, Krieg T (2009). Scleroderma. N Engl J Med.

[9] Siegel RM, Lipsky PE, Firestein GS (2009). Autoimmunity. Kelley's Textbook of Rheumatology.

[10] Shah AA, Rosen A (2011). Cancer and Systemic Sclerosis: Novel Insights into Pathogenesis and Clinical Implications. Curr Opin Rheumatol.

[11] Ostojic P, Cerinic MM, Silver R, Highland K, Damjanov N (2007). Interstitial lung disease in systemic sclerosis. Lung.

[12] Luo Y, Xiao R (2011). Interstitial Lung Disease in Scleroderma: Clinical Features and Pathogenesis. Rheumatology (Oxford).

[13] De Cruz S, Ross D (2013). Lung transplantation in patients with scleroderma. Curr Opin Rheumatol.

[14] Schachna L, Medsger TA, Dauber JH, Wigley FM, Braunstein NA, White B, Steen VD, Conte JV, Yang SC, McCurry KR, Borja MC, Plaskon DE, Orens JB, Gelber AC (2006). Lung transplantation in scleroderma compared with idiopathic pulmonary fibrosis and idiopathic pulmonary arterial hypertension. Arthritis Rheum.

[15] Boin F, De Fanis U, Bartlett SJ, Wigley FM, Rosen A, Casolaro V (2008). T cell polarization identifies distinct clinical phenotypes in scleroderma lung disease. Arthritis Rheum.

[16] Steen VD (1998). Clinical manifestations of systemic sclerosis. Semin Cutan Med Surg.

[17] Lota HK, Renzoni EA (2012). Circulating biomarkers of interstitial lung disease in systemic sclerosis. Int J Rheumatol.

[18] Ludwicka-Bradley A, Silver RM, Bogatkevich GS (2011). Coagulation and autoimmunity in scleroderma interstitial lung disease. Semin Arthritis Rheum.

[19] Whitfield ML, Finlay DR, Murray JI, Troyanskaya OG, Chi JT, Pergamenschikov A, McCalmont TH, Brown PO, Botstein D, Connolly MK (2003). Systemic and cell type-specific gene expression patterns in scleroderma skin. Proc Natl Acad Sci U S A.

[20] Chung L, Utz PJ (2004). Antibodies in scleroderma: direct pathogenicity and phenotypic associations. Curr Rheumatol Rep.

[21] Warrington KJ, Nair U, Carbone LD, Kang AH, Postlethwaite AE (2006). Characterisation of the immune response to type I collagen in scleroderma. Arthritis Res Ther.

[22] Salamunić I (2010). Laboratory diagnosis of autoimmune diseases – new technologies, old dilemmas. Biochemia Medica.

[23] Tashkin DP, Elashoff D, Roth MD, Furst DE, Khanna D, Clements P. Predictors of Change in % Predicted FVC over Time in Scleroderma (SSc) Interstitial Lung Disease (ILD): Findings from the Scleroderma Lung Study (SLS). Am J Respir Crit Care Med. 2009;179.

[24] Perez Campos D, Estevez Del Toro M, Pena Casanovas A, Gonzalez Rojas PP, Morales Sanchez L, Gutierrez Rojas AR (2012). Are high doses of prednisone necessary for treatment of interstitial lung disease in systemic sclerosis?. Reumatol Clin.

[25] Tan A, Denton CP, Mikhailidis DP, Seifalian AM (2011). Recent advances in the diagnosis and treatment of interstitial lung disease in systemic sclerosis (scleroderma): a review. Clin Exp Rheumatol.

[26] Hastie T, Tibsirani R, Friedman J, The Elements of Statistical Learning. Springer Series in Statistics, ed. Springer. New York, Philadelphia; 2015.

[27] Lung function testing: selection of reference values and interpretative strategies. American Thoracic Society. Am Rev Respir Dis. 1991. 144(5): 1202–18 10.1164/ajrccm/144.5.1202 1952453

[28] Hankinson JL, Odencrantz JR, Fedan KB (1999). Spirometric reference values from a sample of the general U.S. population. Am J Respir Crit Care Med.

[29] Knudson RJ, Kaltenborn WT, Knudson DE, Burrows B (1987). The single-breath carbon monoxide diffusing capacity. Reference equations derived from a healthy nonsmoking population and effects of hematocrit. Am Rev Respir Dis.

[30] Morgan C, Knight C, Lunt M, Black CM, Silman AJ (2003). Predictors of end stage lung disease in a cohort of patients with scleroderma. Ann Rheum Dis.

[31] Plastiras SC, Karadimitrakis SP, Ziakas PD, Vlachoyiannopoulos PG, Moutsopoulos HM, Tzelepis GE (2006). Scleroderma lung: Initial forced vital capacity as predictor of pulmonary function decline. Arthritis Rheumatism-Arthritis Care Res.

[32] Shadly SA, Johnson SR, Meaney C, Chau C, Marras TK (2014). Lung Function and Survival in Systemic Sclerosis Interstitial Lung Disease. J Rheumatol.

[33] Simeon CP, Armadans L, Fonollosa V, Solans R, Selva A, Villar M, Lima J, Vaque J, Vilardell M (2003). Mortality and prognostic factors in Spanish patients with systemic sclerosis. Rheumatology (Oxford).

[34] Shapiro HM (2003). Practical flow cytometry.

[35] Picot J, Guerin CL, Le Van Kim C, Boulanger CM (2012). Flow cytometry: retrospective, fundamentals and recent instrumentation. Cytotechnology.

[36] Truchetet ME, Brembilla NC, Montanari E, Chizzolini C (2010). T cell Subsets in Scleroderma Patients. Expert Rev Dermatol.

[37] Perfetto SP, Chattopadhyay PK, Roederer M (2004). Seventeen-colour flow cytometry: unravelling the immune system. Nat Rev Immunol.

[38] Hedley DW, Friedlander ML, Taylor IW, Rugg CA, Musgrove EA (1983). Method for Analysis of Cellular DNA Content of Paraffin-Embedded Pathological Material Using Flow Cytometry. J Histochem Cytochem.

[39] Nicoletti I, Migliorati G, Pagliacci MC, Grignani F, Riccardi C (1991). A rapid and simple method for measuring thymocyte apoptosis by propidium iodide staining and flow cytometry. J Immunol Methods.

[40] Vermes I, Haanen C, Reutelingsperger C (2000). Flow cytometry of apoptotic cell death. J Immunol Methods.

[41] Raja KR, Plasil M, Rihova L, Pelcova J, Adam Z, Hajek R (2014). Flow cytometry-based enumeration and functional characterization of CD8 T regulatory cells in patients with multiple myeloma before and after lenalidomide plus dexamethasone treatment. Cytometry B Clin Cytom.

[42] Breiman L (1984). Classification and Regression Trees.

[43] Breiman L (2001). Random Forests. Mach Learn.

[44] Hothorn T, Hornik K, Zeileis A (2006). Unbiased recursive partitioning: A conditional inference framework. J Comput Graph Stat.

[45] Gregorutti B, Michel BB, Saint-Pierre P (2013). Correlation and variable importance in random forests. arXiv.

[46] Toloşi L, Lengauer T (2011). Classification with correlated features: unreliability of feature ranking and solutions. Bioinformatics.

[47] Strobl C, Boulesteix AL, Kneib T, Augustin T, Zeileis A. Conditional variable importance for random forests. BMC Bioinformatics. 2008;9. 10.1186/1471-2105-9-307 PMC2491635 18620558

[48] Cortes C, Vapnik V (1995). Support-Vector Networks. Mach Learn.

[49] Hastie, T., R. Tibshirani, and J.H. Friedman, The elements of statistical learning : data mining, inference, and prediction. 2nd ed. Springer series in statistics (2009). New York.

[50] Kecman V (2005). Support Vector Machines – An Introduction, in Support vector machines: theory and applications.

[51] Wold S, Esbensen K, Geladi P (1987). Principal Component Analysis. Chemom Intell Lab Syst.

[52] Warnock DG, Peck CC (2010). A roadmap for biomarker qualification. Nat Biotechnol.

[53] Olsen DL, Delen D (2008). Advanced Data Mining Techniques.

[54] Navrac N (1999). Selected Techniques for Data Mining in Medicine. Artif Intell Med.

[55] Zweig MH, Campbell G (1993). Receiver-operating characteristic (ROC) plots: a fundamental evaluation tool in clinical medicine. Clin Chem.

[56] Mootha VK, Lindgren CM, Eriksson KF, Subramanian A, Sihag S, Lehar J, Puigserver P, Carlsson E, Ridderstrale M, Laurila E, Houstis N, Daly MJ, Patterson N, Mesirov JP, Golub TR, Tamayo P, Spiegelman B, Lander ES, Hirschhorn JN, Altshuler D, Groop LC (2003). PGC-1alpha-responsive genes involved in oxidative phosphorylation are coordinately downregulated in human diabetes. Nat Genet.

[57] Subramanian A, Tamayo P, Mootha VK, Mukherjee S, Ebert BL, Gillette MA, Paulovich A, Pomeroy SL, Golub TR, Lander ES, Mesirov JP (2005). Gene set enrichment analysis: a knowledge-based approach for interpreting genome-wide expression profiles. Proc Natl Acad Sci U S A.

[58] R Core Team, A language and environment for statistical computing. R Foundation for Statistical Computing, 2013: p. http://www.R-project.org/.

[59] Dobbin KK, Simon RM (2011). Optimally splitting cases for training and testing high dimensional classifiers. BMC Med Genom.

[60] Strobl C, Hothorn T, Zeileis A. Party on ! A New, Conditional Variable Importance Measure for Random Forests Available in the Party Package Party on. R J Animal Ecol. 2009;050.

[61] Nicodemus KK, Malley JD, Strobl CC, Ziegler A. The behaviour of random forest permutation-based variable importance measures under predictor correlation. BMC Bioinformatics. 2010. 11(1):1-13. 10.1186/1471-2105-11-110 PMC2848005 20187966

[62] Vetter JS. Contemporary high performance computing: from Petascale toward exascale. New York: Taylor & Francis; 2013.

[63] Orre R, Lansner A, Bate A, Lindquist M (2000). Bayesian neural networks with confidence estimations applied to data mining. Computational Stat Data Anal.

[64] Lisboa PJG, Wong H, Harris P, Swindell R (2003). A Bayesian neural network approach for modelling censored data with an application to prognosis after surgery for breast cancer. Artif Intell Med.

[65] Luo WJ, Friedman MS, Shedden K, Hankenson KD, Woolf PJ. GAGE: generally applicable gene set enrichment for pathway analysis. BMC Bioinformatics. 2009. 10:1-17 10.1186/1471-2105-10-161 PMC2696452 19473525

[66] Barry WT, Nobel AB, Wright FA (2005). Significance analysis of functional categories in gene expression studies: a structured permutation approach. Bioinformatics.

[67] Dinu I, Potter JD, Mueller T, Liu Q, Adewale AJ, Jhangri GS, et al. Improving gene set analysis of microarray data by SAM-GS. BMC Bioinformatics. 2007;8. 10.1186/1471-2105-8-242 PMC1931607 17612399

[68] Kim SY, Volsky DJ. PAGE: Parametric analysis of gene set enrichment. BMC Bioinformatics. 2005;6. 10.1186/1471-2105-6-144 PMC1183189 15941488

[69] Boorsma A, Foat BC, Vis D, Klis F, Bussemaker HJ (2005). T-profiler: scoring the activity of predefined groups of genes using gene expression data. Nucleic Acids Res.

[70] Newton MA, Quintana FA, Den Boon JA, Sengupta S, Ahlquist P (2007). Random-Set Methods Identify Distinct Aspects of the Enrichment Signal in Gene-Set Analysis. Ann Appl Stat.

