# Supplementary material for: A methodology for exploring biomarker – phenotype associations: application to flow cytometry data and systemic sclerosis clinical manifestations
Source: BMC Bioinformatics. 2015 Sep 15;16:293. doi: 10.1186/s12859-015-0722-x (PMC4571079; doi:10.1186/s12859-015-0722-x)
Supplement: Additional file 1: — Flow Cytometry Details and Supplementary Results. (DOCX 1.93 MB) [file 12859_2015_722_MOESM1_ESM.docx]

**Flow Cytometry Details and Supplementary Results**

The FCS data, detailed description of the gating strategy and description of antibodies and fluorochromes can be found at <https://flowrepository.org/id/FR-FCM-ZZLH>

**Section 1 - Flow Cytometry**

The 112 T cell flow cytometry variables contained in the data set (IRIS_MASTER.csv) are grouped into 4 functional panels: activation, memory, polarization and trafficking. Each functional panel has different T cell subsets that are connected to each other through a hierarchical structure shown on CD4 cells for convenience. Included herein for each panel are the flow cytometry gating strategies (Figures 1-1 to 1-4) and the logical hierarchical views (Figures 1-5 to 1-8). All of the child nodes are expressed as percent of the parent node. These subset T cells were named using codes whose definitions can be found in Table 1-1. All the panels share the same high order gating strategy to identify CD4^+^ and CD8^+^ T cells by using the lineage markers CD3, CD4 and CD8. Each T cell population was then divided into sub-categories specific to each panel. In the memory panel, CD45 RA and CCR7 were used to identify central memory T cell, effector memory T cell and effector memory CD45RA^+^ cells. CD27 and CD28 were used to subgroup memory populations. CD57 was used as marker of terminally differentiated memory cells. Naïve T cells were defined by the expression of CD45RA, CCR7, CD27 and CD28. In the polarization panel CCR4, CCR6 and CXR3 were used to identify Th1, Th1/Th17, Th2 and Th17 helper cells. CRTh2 was also used as marker of Th2 cells, while CCR5 was used as a marker of Th1. In the activation panel, CD69, HLA-DR and CD71 were used to evaluate early and mid-early T cell activation. CD25 low is a marker of T cell activation, while CD25 high and CD103 are associated with regulatory T cells. In the trafficking panel, CCR2 was used as marker of gut homing, CCR3 as marker of upper airway homing, CCR10 as marker of skin homing, CXCR4 as marker of T cell homeostasis, CXCR5 as marker of circulating T follicular helper cells and CXCR6 as marker of activated and lung antigen specific T cells. In Tables 1-2 to 1-5 the antibodies and fluorochromes used to characterize PBMC are listed.

**Figure 1-1 Activation Panel**

**Activation panel gating strategy**

(A) Lymphocytes were identified on the base of their forward (FSC-A) and side scatter (SSC-A). T lymphocytes were gated based on the expression of the T cell receptor (CD3) and then sub-gated in CD4^+^/CD8^-^ and CD4^-^/CD8^+^ T cells. Density plot representation of HLA-DR, CD69, CD25, CD71 and CD103 expression on CD8^+^ and CD4^+^ T cells.

(B) Populations identified by Boolean combination of T cell activation markers.

| act42569 | CD3+/CD4+/CD8-/CD25tot/CD69+ |
| --- | --- |
| act42571 | CD3+/CD4+/CD8-/CD25tot/CD71+ |
| act425103 | CD3+/CD4+/CD8-/CD25tot/CD103+ |
| act425hladr | CD3+/CD4+/CD8-/CD25tot/HLADR+ |
| act46971 | CD3+/CD4+/CD8-/CD69+/CD71+ |
| act469hladr | CD3+/CD4+/CD8-/CD69/HLADR+ |
| act410371 | CD3+/CD4+/CD8-/CD103+/CD71+ |
| act4103hladr | CD3+/CD4+/CD8-/CD103+/HLADR+ |
| act471hladr | CD3+/CD4+/CD8-/CD71+/HLADR+ |
| act82571 | CD3+/CD4-/CD8+/CD25tot/CD71+ |
| act86925 | CD3+/CD4-/CD8+/CD69+/CD25tot |
| act86971 | CD3+/CD4-/CD8+/CD69+/CD71+ |
| act869hladr | CD3+/CD4-/CD8+/CD69+/HLADR+ |
| act810325 | CD3+/CD4-/CD8+/CD103+/CD25tot |
| act810369 | CD3+/CD4-/CD8+/CD103+/CD69+ |
| act8103hladr | CD3+/CD4-/CD8+/CD103+/HLADR+ |
| act810371 | CD3+/CD4-/CD8+/CD103+/CD71+ |
| act8hladr25 | CD3+/CD4-/CD8+/HLADR+/CD25tot |
| act8hladr71 | CD3+/CD4-/CD8+/HLADR+/CD71+ |
| act42569 | CD3+/CD4+/CD8-/CD25tot/CD69+ |

**Figure 1-2 Memory Panel**

**Memory panel gating strategy**

(A) Lymphocytes were identified on the base of their forward (FSC-A) and side scatter (SSC-A). T lymphocytes were gated based on the expression of the T cell receptor (CD3) and then sub-gated in CD4^+^ and CD8^+^ T cells. Density plot representation of CD27, CD28 and CD57 expression on CD8^+^ and CD4^+^ T cells. Central memory(CM), effector memory (EM) and effector memory CD45RA positive cells (EMRA) were identified by CD45RA and CCR7 expression. Naive T cells were identified by using CD45RA, CCR7, CD27 and CD28.

(B) Populations identified by Boolean combination of T cell memory markers.

| mememra478 | CD3+/CD4+/CD8-/CD45RA+/CCR7-/CD27+/CD28+ |
| --- | --- |
| mememra47 | CD3+/CD4+/CD8-/CD45RA+/CCR7-/CD27+/CD28- |
| mememra48 | CD3+/CD4+/CD8-/CD45RA+/CCR7-/CD27-/CD28+ |
| mememra40 | CD3+/CD4+/CD8-/CD45RA+/CCR7-/CD27-/CD28- |
| memcm478 | CD3+/CD4+/CD8-/CD45RA-/CCR7+/CD27+/CD28+ |
| memcm47 | CD3+/CD4+/CD8-/CD45RA-/CCR7+/CD27+/CD28- |
| memcm48 | CD3+/CD4+/CD8-/CD45RA-/CCR7-/CD27-/CD28+ |
| memcm40 | CD3+/CD4+/CD8-/CD45RA-/CCR7+/CD27-/CD28- |
| memem478 | CD3+/CD4+/CD8-/CD45RA-/CCR7-/CD27+/CD28- |
| memem47 | CD3+/CD4+/CD8-/CD45RA-/CCR7-/CD27+/CD28- |
| memem48 | CD3+/CD4+/CD8-/CD45RA-/CCR7-/CD27-/CD28+ |
| memem40 | CD3+/CD4+/CD8-/CD45RA-/CCR7-/CD27-/CD28- |
| memcm4k | CD3+/CD4+/CD8-/CD45RA-/CCR7+/CD57+ |
| mememra4k | CD3+/CD4+/CD8-/CD45RA+/CCR7-/CD57+ |
| memem4k | CD3+/CD4+/CD8-/CD45RA-/CCR7-/CD57+ |

| mememra878 | CD3+/CD4-/CD8+/CD45RA+/CCR7-/CD27+/CD28+ |
| --- | --- |
| mememra87 | CD3+/CD4-/CD8+/CD45RA+/CCR7-/CD27+/CD28- |
| mememra88 | CD3+/CD4-/CD8+/CD45RA+/CCR7-/CD27-/CD28+ |
| mememra80 | CD3+/CD4-/CD8+/CD45RA+/CCR7-/CD27-/CD28- |
| memcm878 | CD3+/CD4-/CD8+/CD45RA-/CCR7+/CD27+/CD28+ |
| memcm87 | CD3+/CD4-/CD8+/CD45RA-/CCR7+/CD27+/CD28- |
| memcm88 | CD3+/CD4-/CD8+/CD45RA-/CCR7+/CD27-/CD28+ |
| memcm80 | CD3+/CD4-/CD8+/CD45RA-/CCR7+/CD27-/CD28- |
| memem878 | CD3+/CD4-/CD8+/CD45RA-/CCR7-/CD27+/CD28+ |
| memem87 | CD3+/CD4-/CD8+/CD45RA-/CCR7-/CD27+/CD28- |
| memem88 | CD3+/CD4-/CD8+/CD45RA-/CCR7-/CD27-/CD28+ |
| memem80 | CD3+/CD4-/CD8+/CD45RA-/CCR7-/CD27-/CD28- |
| memcm8k | CD3+/CD4-/CD8+/CD45RA-/CCR7+/CD57+ |
| mememra8k | CD3+/CD4-/CD8+/CD45RA+/CCR7-/CD57+ |
| memem8k | CD3+/CD4-/CD8+/CD45RA-/CCR7-/CD57+ |

**Figure 1-3 Polarization Panel**

**Polarization panel gating strategy**

(A) Lymphocytes were identified on the base of their forward (FSC-A) and side scatter (SSC-A). T lymphocytes were gated based on the expression of the T cell receptor (CD3) and then sub-gated in CD4^+^/CD8^-^ and CD4^-^/CD8^+^ T cells. Density plot representation of CXCR3, CRTh2, CCR6, CCR4 and CCR5 expression on CD8^+^ and CD4^+^ T cells. CCR4, CCR6 and CXCR3 were used to identify Th1, Th1/17, Th2, Th17 cells.

(B) Populations identified by Boolean combination of T cell polarization markers.

| pol4ccr5cxcr3 | CD3+/CD4+/CD8-/CCR5+/CXCR3+ |
| --- | --- |
| pol4ccr5cxcr3neg | CD3+/CD4+/CD8-/CCR5+/CXCR3- |
| pol4ccr5negcxcr3 | CD3+/CD4+/CD8-/CCR5-/CXCR3+ |
| pol8ccr5cxcr3 | CD3+/CD4-/CD8+/CCR5+/CXCR3+ |
| pol8ccr5cxcr3neg | CD3+/CD4-/CD8+/CCR5+/CXCR3- |
| pol8ccr5negcxcr3 | CD3+/CD4-/CD8+/CCR5-/CXCR3+ |

**Figure 1-4 Traffic Panel**

**Trafficking panel gating strategy**

(A) Lymphocytes were identified on the base of their forward (FSC-A) and side scatter (SSC-A). T lymphocytes were gated based on the expression of the T cell receptor (CD3) and then sub-gated in CD4^+^/CD8^-^ and CD4^-^/CD8^+^ T cells. Density plot representation of CCR2, CCR3, CCR10, CXCR4, CXCR5 and CXCR6 expression on CD8^+^ and CD4^+^ T cells.


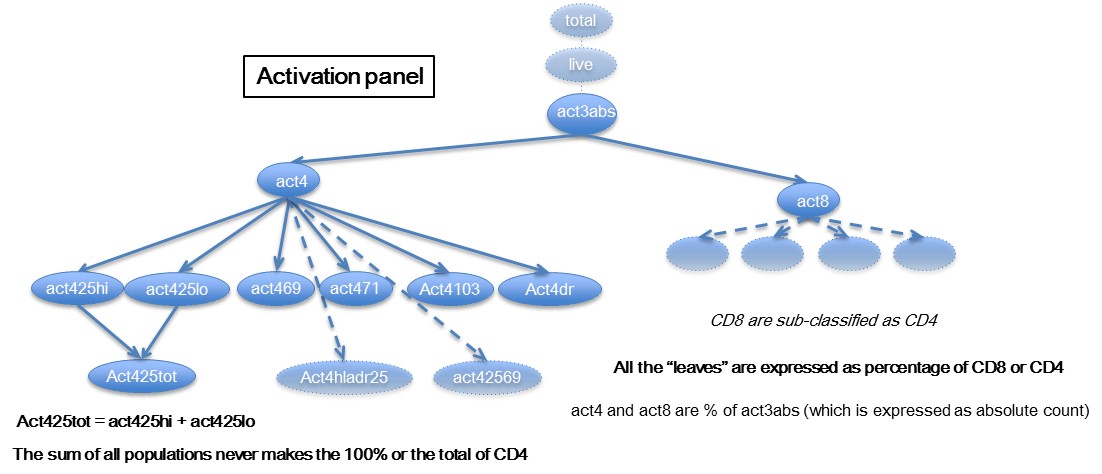
**Figure 1-5 Activation Panel Hierarchy**


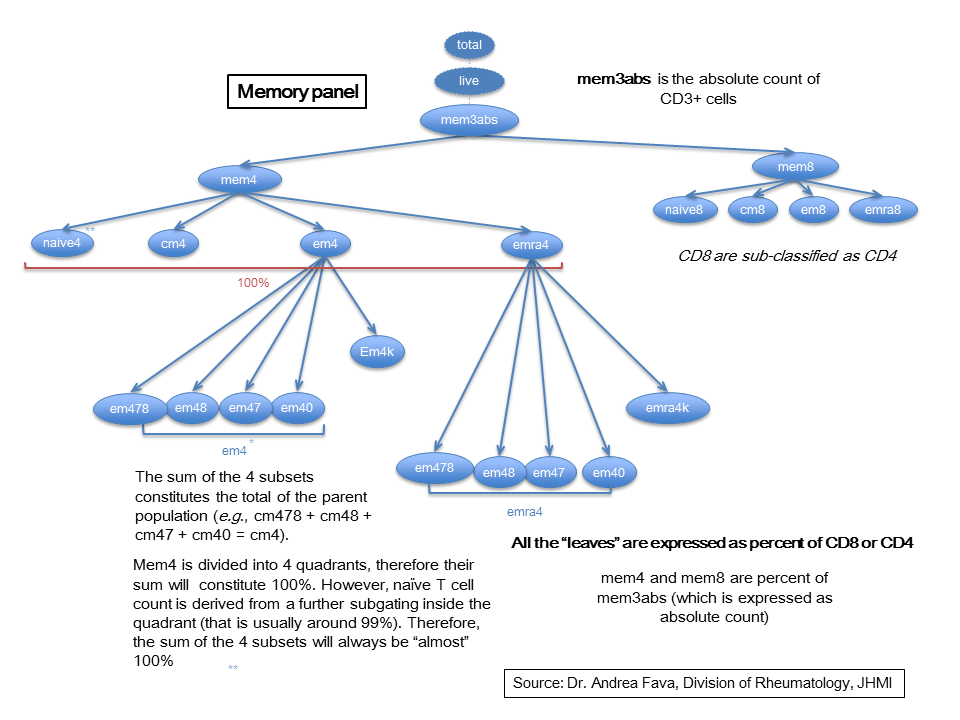
**Figure 1-6 Memory Panel Hierarchy**

**Figure 1-7 Polarization Panel Hierarchy**


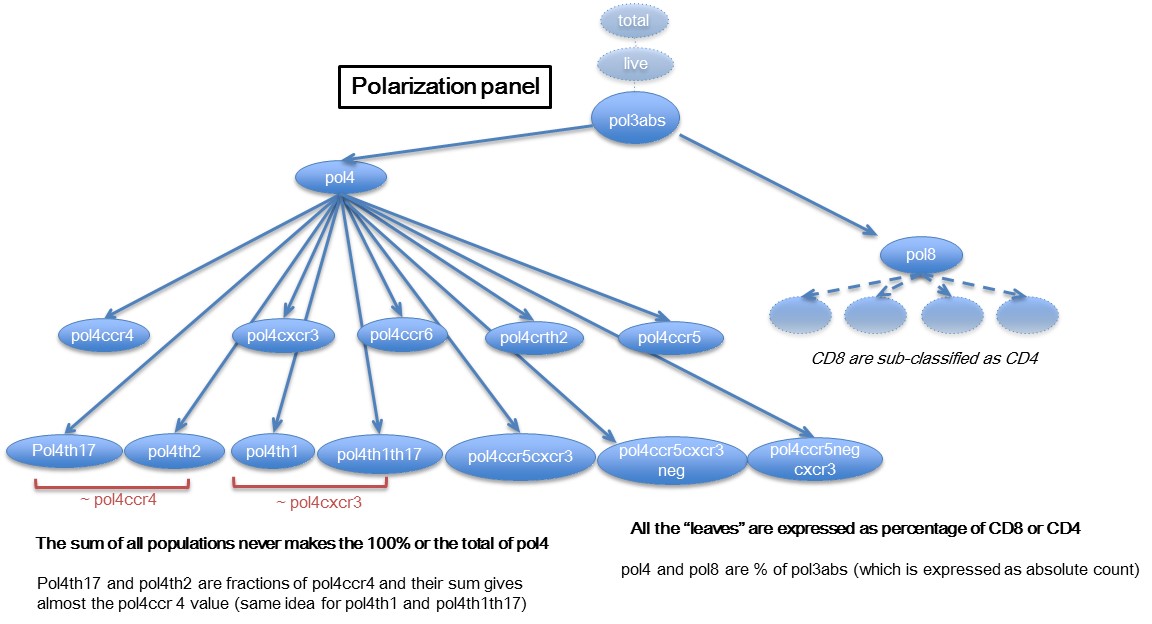


**Figure 1-8 Trafficking Panel Hierarchy**


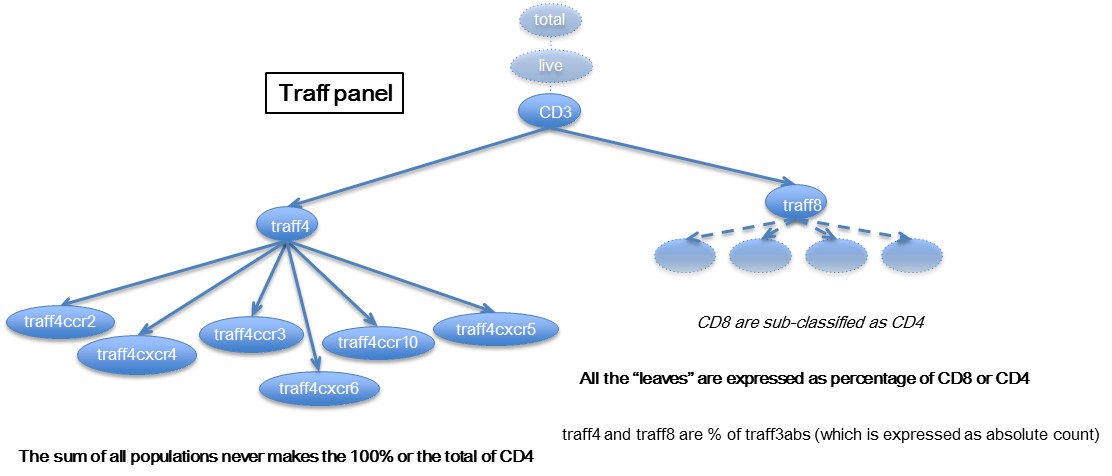


**Table 1-1 Definitions of All Subset T cells**

| **code** | **subset definition** |
| --- | --- |
| pol4 | CD3+/CD4+/CD8- |
| pol8 | CD3+/CD4-/CD8+ |
| pol4ccr4 | CD3+/CD4+/CD8-/CCR4+ |
| pol4ccr5 | CD3+/CD4+/CD8-/CCR5+ |
| pol4ccr6 | CD3+/CD4+/CD8-/CCR6+ |
| pol4crth2 | CD3+/CD4+/CD8-/CRTh2+ |
| pol4cxcr3 | CD3+/CD4+/CD8-/CXCR3+ |
| pol4ccr5cxcr3 | CD3+/CD4+/CD8-/CCR5+/CXCR3+ |
| pol4ccr5cxcr3neg | CD3+/CD4+/CD8-/CCR5+/CXCR3- |
| pol4ccr5negcxcr3 | CD3+/CD4+/CD8-/CCR5-/CXCR3+ |
| pol4th1 | CD3+/CD4+/CD8-/CXCR3+/CCR4-/CCR6- |
| pol4th1th17 | CD3+/CD4+/CD8-/CXCR3+/CCR4-/CCR6+ |
| pol4th2 | CD3+/CD4+/CD8-/CXCR3-/CCR4+/CCR6- |
| pol4th17 | CD3+/CD4+/CD8-/CXCR3-/CCR4+/CCR6+ |
| pol8ccr4 | CD3+/CD4-/CD8+/CCR4+ |
| pol8ccr5 | CD3+/CD4-/CD8+/CCR5+ |
| pol8ccr6 | CD3+/CD4-/CD8+/CCR6+ |
| pol8crth2 | CD3+/CD4-/CD8+/CRTh2+ |
| pol8cxcr3 | CD3+/CD4-/CD8+/CXCR3+ |
| pol8ccr5cxcr3 | CD3+/CD4-/CD8+/CCR5+/CXCR3+ |
| pol8ccr5cxcr3neg | CD3+/CD4-/CD8+/CCR5+/CXCR3- |
| pol8ccr5negcxcr3 | CD3+/CD4-/CD8+/CCR5-/CXCR3+ |
| pol8th1 | CD3+/CD4-/CD8+/CXCR3+/CCR4-/CCR6- |
| pol8th1th17 | CD3+/CD4-/CD8+/CXCR3+/CCR4-/CCR6+ |
| pol8th2 | CD3+/CD4-/CD8+/CXCR3-/CCR4+/CCR6- |
| pol8th17 | CD3+/CD4-/CD8+/CXCR3-/CCR4+/CCR6+ |
| cd4cd8ratioLOG | Log10(ratio of pol4 over pol8) |
| pol8th1th2ratio | ratio of CD8+/CCR5+ to CD8+/CRTh2 |
| pol8x3r4ratio | ratio of CD8+/CXCR3+ to CD8+/CCR4+ |
| cd8r5th2ratio | ratio of CD8+/CCR5+ to CD8+/CRTh2 |
| cd4r5th2ratio | ratio of CD4+/CCR5+ to CD4+/CRTh2 |
| act425hi | CD3+/CD4+/CD8-/CD25hi |
| act425lo | CD3+/CD4+/CD8-/CD25lo |
| act425tot | CD3+/CD4+/CD8-/CD25+ |
| act469 | CD3+/CD4+/CD8-/CD69+ |
| act471 | CD3+/CD4+/CD8-/CD71+ |
| act4103 | CD3+/CD4+/CD8-/CD103+ |
| act4dr | CD3+/CD4+/CD8-/HLADR+ |
| act825 | CD3+/CD4-/CD8+/CD25+ |
| act869 | CD3+/CD4-/CD8+/CD69+ |
| act871 | CD3+/CD4-/CD8+/CD71+ |
| act8103 | CD3+/CD4-/CD8+/CD103+ |
| act8dr | CD3+/CD4-/CD8+/HLADR+ |
| act42569 | CD3+/CD4+/CD8-/CD25tot/CD69+ |
| act42571 | CD3+/CD4+/CD8-/CD25tot/CD71+ |
| act425103 | CD3+/CD4+/CD8-/CD25tot/CD103+ |
| act425hladr | CD3+/CD4+/CD8-/CD25tot/HLADR+ |
| act46971 | CD3+/CD4+/CD8-/CD69+/CD71+ |
| act469hladr | CD3+/CD4+/CD8-/CD69/HLADR+ |
| act410371 | CD3+/CD4+/CD8-/CD103+/CD71+ |
| act4103hladr | CD3+/CD4+/CD8-/CD103+/HLADR+ |
| act471hladr | CD3+/CD4+/CD8-/CD71+/HLADR+ |
| act82571 | CD3+/CD4-/CD8+/CD25tot/CD71+ |
| act86925 | CD3+/CD4-/CD8+/CD69+/CD25tot |
| act86971 | CD3+/CD4-/CD8+/CD69+/CD71+ |
| act869hladr | CD3+/CD4-/CD8+/CD69+/HLADR+ |
| act810325 | CD3+/CD4-/CD8+/CD103+/CD25tot |
| act810369 | CD3+/CD4-/CD8+/CD103+/CD69+ |
| act8103hladr | CD3+/CD4-/CD8+/CD103+/HLADR+ |
| act810371 | CD3+/CD4-/CD8+/CD103+/CD71+ |
| act8hladr25 | CD3+/CD4-/CD8+/HLADR+/CD25tot |
| act8hladr71 | CD3+/CD4-/CD8+/HLADR+/CD71+ |
| traff4ccr2 | CD3+/CD4+/CD8-/CCR2+ |
| traff4ccr3 | CD3+/CD4+/CD8-/CCR3+ |
| traff4ccr10 | CD3+/CD4+/CD8-/CCR10+ |
| traff4cxcr5 | CD3+/CD4+/CD8-/CXCR5+ |
| traff8ccr2 | CD3+/CD4-/CD8+/CCR2+ |
| traff8ccr3 | CD3+/CD4-/CD8+/CCR3+ |
| traff8ccr10 | CD3+/CD4-/CD8+/CCR10+ |
| traff8cxcr5 | CD3+/CD4-/CD8+/CXCR5+ |
| traff4cxcr4 | CD3+/CD4+/CD8-/CXCR4+ |
| traff4cxcr6 | CD3+/CD4+/CD8-/CXCR6+ |
| traff8cxcr4 | CD3+/CD4-/CD8+/CXCR4+ |
| traff8cxcr6 | CD3+/CD4-/CD8+/CXCR6+ |
| memnaive4 | CD3+/CD4+/CD8-/CD45RA+/CCR7+/CD27+/CD28+ (Naïve T cells) |
| memcm4 | CD3+/CD4+/CD8-/CD45RA-/CCR7+ (Central memory) |
| mememra4 | CD3+/CD4+/CD8-/CD45RA+/CCR7- (Terminally differentiated "effector memory CD45RA+" cells) |
| memem4 | CD3+/CD4+/CD8-/CD45RA-/CCR7- (effector memory) |
| mememra478 | CD3+/CD4+/CD8-/CD45RA+/CCR7-/CD27+/CD28+ |
| mememra47 | CD3+/CD4+/CD8-/CD45RA+/CCR7-/CD27+/CD28- |
| mememra48 | CD3+/CD4+/CD8-/CD45RA+/CCR7-/CD27-/CD28+ |
| mememra40 | CD3+/CD4+/CD8-/CD45RA+/CCR7-/CD27-/CD28- |
| memcm478 | CD3+/CD4+/CD8-/CD45RA-/CCR7+/CD27+/CD28+ |
| memcm47 | CD3+/CD4+/CD8-/CD45RA-/CCR7+/CD27+/CD28- |
| memcm48 | CD3+/CD4+/CD8-/CD45RA-/CCR7-/CD27-/CD28+ |
| memcm40 | CD3+/CD4+/CD8-/CD45RA-/CCR7+/CD27-/CD28- |
| memem478 | CD3+/CD4+/CD8-/CD45RA-/CCR7-/CD27+/CD28- |
| memem47 | CD3+/CD4+/CD8-/CD45RA-/CCR7-/CD27+/CD28- |
| memem48 | CD3+/CD4+/CD8-/CD45RA-/CCR7-/CD27-/CD28+ |
| memem40 | CD3+/CD4+/CD8-/CD45RA-/CCR7-/CD27-/CD28- |
| memcm4k | CD3+/CD4+/CD8-/CD45RA-/CCR7+/CD57+ |
| mememra4k | CD3+/CD4+/CD8-/CD45RA+/CCR7-/CD57+ |
| memem4k | CD3+/CD4+/CD8-/CD45RA-/CCR7-/CD57+ |
| memnaive8 | CD3+/CD4-/CD4-/CD8+/CD45RA+/CCR7+/CD27+/CD28+ (Naïve T cells) |
| memcm8 | CD3+/CD4-/CD4-/CD8+/CD45RA-/CCR7+ (Central memory) |
| mememra8 | CD3+/CD4-/CD8+/CD45RA+/CCR7- (Terminally differentiated "effector memory CD45RA+" cells) |
| memem8 | CD3+/CD4-/CD8+/CD45RA-/CCR7- (effector memory) |
| mememra878 | CD3+/CD4-/CD8+/CD45RA+/CCR7-/CD27+/CD28+ |
| mememra87 | CD3+/CD4-/CD8+/CD45RA+/CCR7-/CD27+/CD28- |
| mememra88 | CD3+/CD4-/CD8+/CD45RA+/CCR7-/CD27-/CD28+ |
| mememra80 | CD3+/CD4-/CD8+/CD45RA+/CCR7-/CD27-/CD28- |
| memcm878 | CD3+/CD4-/CD8+/CD45RA-/CCR7+/CD27+/CD28+ |
| memcm87 | CD3+/CD4-/CD8+/CD45RA-/CCR7+/CD27+/CD28- |
| memcm88 | CD3+/CD4-/CD8+/CD45RA-/CCR7+/CD27-/CD28+ |
| memcm80 | CD3+/CD4-/CD8+/CD45RA-/CCR7+/CD27-/CD28- |
| memem878 | CD3+/CD4-/CD8+/CD45RA-/CCR7-/CD27+/CD28+ |
| memem87 | CD3+/CD4-/CD8+/CD45RA-/CCR7-/CD27+/CD28- |
| memem88 | CD3+/CD4-/CD8+/CD45RA-/CCR7-/CD27-/CD28+ |
| memem80 | CD3+/CD4-/CD8+/CD45RA-/CCR7-/CD27-/CD28- |
| memcm8k | CD3+/CD4-/CD8+/CD45RA-/CCR7+/CD57+ |
| mememra8k | CD3+/CD4-/CD8+/CD45RA+/CCR7-/CD57+ |
| memem8k | CD3+/CD4-/CD8+/CD45RA-/CCR7-/CD57+ |

**Tables 1-2 to 1-5: antibodies and fluorochromes used to characterize PBMC**

| **Table 1-2 Activation Panel** | | | |
| --- | --- | --- | --- |
| **Antibody** | **Fluorochrome** | **Clone** | **Company** |
| CD69 | FITC | FN50 | BD Pharmingen |
| CD103 | PE | Ber-ACT8 | BioLegend |
| HLA-DR | PE-TR | TÜ36 | Invitrogen |
| CD71 | PE-Cy5 | M-A712 | BD Pharmingen |
| CD25 | PE-Cy7 | 2A3 | BD |
| CD8 | APC-H7 | SK1 | BD |
| CD4 | Pacific Blue | RPA-T4 | BD |
| CD3 | BV510 | OKT3 | BioLegend |

| **Table 1-3 Memory Panel** | | | |
| --- | --- | --- | --- |
| **Antibody** | **Fluorochrome** | **Clone** | **Company** |
| CD27 | FITC | M-T271 | BD |
| CD28 | PE | CD28.2 | BD |
| CD45RA | PE-TR | MEM-56 | Invitrogen |
| CCR7 | PerCP/Cy5.5 | G043H7 | BioLegend |
| CD57 | APC | HCD57 | BioLegend |
| CD8 | APC-H7 | SK1 | BD |
| CD4 | Pacific Blue | RPA-T4 | BD |
| CD3 | BV510 | OKT3 | BioLegend |

| **Table 1-4 Polarization Panel** | | | |
| --- | --- | --- | --- |
| **Antibody** | **Fluorochrome** | **Clone** | **Company** |
| CXCR3 | Alexa 488 | G025H7 | BioLegend |
| CRTH2 | PE | BM16 | Miltenyi |
| CCR6 | PerCP/Cy5.5 | G034E3 | BioLegend |
| CCR4 | PE-Cy7 | 1G1 | BD |
| CCR10 | APC | 314305 | R&D |
| CCR5 | A700 | HEK/1/85a | BioLegend |
| CD8 | APC-H7 | SK1 | BD |
| CD4 | Pacific Blue | RPA-T4 | BD |
| CD3 | BV510 | OKT3 | BioLegend |

| **Table 1-5 Trafficking Panel** | | | |
| --- | --- | --- | --- |
| **Antibody** | **Fluorochrome** | **Clone** | **Company** |
| CXCR5 | FITC | 51505 | R&D |
| CCR3 | PE | 5E8 | BD Pharmingen |
| CCR2 | PerCP/Cy5.5 | K036C2 | BioLegend |
| CXCR4 | PE-Cy7 | 12G5 | Biolegend |
| CXCR6 | APC | 56811 | R&D |
| CD8 | APC-H7 | SK1 | BD |
| CD4 | Pacific Blue | RPA-T4 | BD |
| CD3 | BV510 | OKT3 | BioLegend |

**Flow Cytometry Online References**

<http://onlinelibrary.wiley.com/doi/10.1002/cyto.a.20643/full>

<http://onlinelibrary.wiley.com/doi/10.1034/j.1399-0039.1999.540606.x/abstract>

<http://onlinelibrary.wiley.com/doi/10.1002/1521-4141(200010)30:10%3C2972::AID-IMMU2972%3E3.0.CO;2-%23/abstract>

<http://www.nature.com/ni/journal/v8/n6/full/ni1467.html>

<http://www.direct-ms.org/pdf/ImmunityRegulation/Tolerance%20to%20self%20Nature%20Symp.pdf>

<http://www.jimmunol.org/content/174/9/5444.long>

<http://jem.rupress.org/content/202/8/1051.full>

<http://www.sciencedirect.com/science/article/pii/S0022175904002571>

<http://www.nature.com/mi/journal/v2/n6/full/mi2009105a.html>

<http://www.nature.com/nri/journal/v8/n2/full/nri2236.html>

<http://www.nature.com/mi/journal/v8/n1/full/mi201446a.html#close>

<http://www.jimmunol.org/content/169/3/1189.long>

<http://www.ncbi.nlm.nih.gov/pmc/articles/PMC1774196/>

<http://www.sciencedirect.com/science/article/pii/S1074761310004917>

<http://iai.asm.org/content/79/8/3328.full>

**Section 2 - Best Training Data Set Screening Tools**

Following in Table 2-1 are details of the best training screening tools for no pre-partitioning (Level 0) and all lower levels. The FC variable names are shown as are the corresponding standardized random threshold deviates.

| Variable/Node | 0 | 1-1 | 1-2 | 2-1 | 2-2 | 3-1 | 3-2 | 4-2 | 4-3 |
| --- | --- | --- | --- | --- | --- | --- | --- | --- | --- |
| act4103 | 0.57 |  |  |  | -1.58 |  |  |  |  |
| act425lo |  | 2.10 | -1.60 |  | -1.63 |  | 1.82 |  |  |
| act425tot | 2.29 |  | 0.07 |  | -0.24 |  |  | 2.47 |  |
| act8103 |  |  |  |  |  |  |  |  | 1.17 |
| act810371 | 1.43 | 2.44 | 0.68 | 2.13 |  |  | 1.28 |  | 2.09 |
| memem4 | 1.05 | 0.98 |  |  | -0.59 |  |  |  |  |
| memem478 |  |  |  |  | -0.41 | 1.85 |  |  | -1.07 |
| memem48 |  |  | -0.27 |  |  | 2.34 | 1.31 | 1.81 | 0.70 |
| memcm478 |  |  |  |  |  |  |  |  | -0.26 |
| memcm4k |  |  |  |  |  | 3.12 |  |  |  |
| memem8 |  |  |  | -1.26 |  |  |  |  |  |
| memem878 | -1.23 | -0.92 |  |  |  |  |  |  |  |
| mememra4 |  |  |  |  |  |  |  | 1.88 |  |
| mememra4k |  |  |  |  |  | 2.82 |  |  |  |
| mememra478 |  |  |  |  |  |  |  | 3.46 |  |
| pol8th17 |  |  | 1.84 |  |  |  | -0.68 | 1.26 |  |
| pol8th1th2ratio |  |  |  | 1.64 |  | 1.67 |  |  |  |
| pol4ccr6 |  | -1.33 |  | -1.36 |  |  |  |  |  |
| traff4ccr3 | 1.88 |  |  | 2.31 |  |  | 0.61 |  |  |

Of the full set of 27 “best FC set” variables, 19 appeared in the highest performing training screening tools. Activation and polarization variables have the highest representation. Only three variables, act425tot, act810371 and memem4 appeared in all Levels (0-4). Pre-partitioning had very pronounced effects on active variables within and across levels. In Level 2 for example, node 2-2 FC thresholds are all negative (cutoff thresholds for those variables less than their means). For all levels, active variable sets for all nodes are typically very different.

**Section 3 - Best Validation Data Set Screening Tools**

Table 3-1 gives the details of the best validation filters. Note that the best training filters are not the best validation filters.

| Variable/Node | 0 | 1-1 | 1-2 | 2-1 | 2-2 | 3-1 | 3-2 | 4-2 | 4-3 |
| --- | --- | --- | --- | --- | --- | --- | --- | --- | --- |
| act4103 |  |  | -0.08 | -0.08 | -1.21 |  |  |  |  |
| act425lo | 1.26 |  | 1.30 | 1.30 |  | 0.65 |  | 1.85 |  |
| act425tot |  |  | 1.04 | 1.04 | -1.45 | 0.48 |  | 1.89 | 1.62 |
| act8103 | 3.23 |  |  |  | 0.06 |  |  |  |  |
| act810371 |  | 2.69 |  |  |  |  | 1.05 |  |  |
| memem4 | 3.59 | 1.54 |  |  |  |  |  |  |  |
| memem478 |  |  |  |  |  |  |  |  | 0.65 |
| memem48 | 3.94 |  |  |  |  |  | 1.83 |  |  |
| memcm478 |  |  |  |  |  | -1.33 | 1.05 |  |  |
| memem8 |  | -0.87 | 0.23 |  |  |  |  |  |  |
| memem878 |  |  |  |  |  |  |  | -0.97 | 2.05 |
| mememra4 |  |  |  |  | 1.14 |  | 5.22 | 1.00 |  |
| mememra4k |  | 6.50 |  |  |  |  |  |  |  |
| pol8ccr4 |  |  |  | 0.23 |  |  |  |  |  |
| pol8th2 |  |  |  |  |  | 1.95 | -0.68 |  |  |
| pol8th17 |  |  | 2.84 | 2.84 |  |  |  |  | 2.48 |
| pol8x3r4ratio |  | 2.60 |  |  |  | -1.28 |  |  |  |
| traff4ccr3 |  |  |  |  | 0.29 |  |  | 0.91 | -0.11 |

Only 18 of the 27 “best FC set” variables were used in the best performing validation filters. All act4103 variables that were active in establishing ILD status had thresholds below their mean. On occasion we see thresholds that correspond to extremely high FC expressions (e.g., 5.22 standard deviations above the mean for mememra4; 6.50 for mememra4k).

**Section 4-1 – Master R code**

We have provided a file “*MachineLearning_GSEA_ScreeningTool.r”* that contains R code to:

1. Execute different machine learning methods

2. Use output from (1) and perform Gene Set Enrichment Analysis (GSEA)

3. Train random filters based on the output of GSEA

4. Perform validation for the best performing screening tools

**Additional Guidance on screening tool execution (training and validation)**

A very large number of random realizations are necessary to produce screening tools with good OMR performance (i.e., most randomly generated tools have poor OMR performance). This required the simultaneous execution of many R instances for training data set screening tool design because of individual compute node execution time limits on Kraken (24 hour wall clock time). An example of an output file from one such instance is provided in the file

**OMR.FC27_0-27choose6.66049.18495142.txt**

Output files are differentiated from one another by simply giving each file a name that contains a randomly generated sequence of numbers (i.e., 66049.18495142). For an entire analysis, the R code “*FC27_0-27choose6.load.r”* concatenates all the relevant txt output files (the output from 718 individual R instances). A non-pre-partitioned concatenated result is provided in the file

**OMR.MASTER.FC27_0-27choose.txt**

Pre-partitioned concatenated results are found in:

**OMR.MASTER.FC27_1-1.txt**

**OMR.MASTER.FC27_1-2.txt**

**OMR.MASTER.FC27_2-1.txt**

**OMR.MASTER.FC27_2-2.txt**

**OMR.MASTER.FC27_2-3.txt**

**OMR.MASTER.FC27_3-1.txt**

**OMR.MASTER.FC27_3-2.txt**

**OMR.MASTER.FC27_3-3.txt**

**OMR.MASTER.FC27_3-4.txt**

**OMR.MASTER.FC27_4-1.txt**

**OMR.MASTER.FC27_4-2.txt**

**OMR.MASTER.FC27_4-3.txt**

**OMR.MASTER.FC27_4-4.txt**

**OMR.MASTER.FC27_4-5.txt**

The entries in each instance file and accordingly in the concatenated file are not sorted (by OMR for example). For all of the concatenated files, the best training screening tools were extracted simply through grep, with the results copied to an excel spreadsheet.
